# Role of myeloid cell leptin signaling in the regulation of glucose metabolism

**DOI:** 10.1038/s41598-021-97549-0

**Published:** 2021-09-15

**Authors:** Sandra Pereira, Daemon L. Cline, Melissa Chan, Kalin Chai, Ji Soo Yoon, Shannon M. O’Dwyer, Cara E. Ellis, Maria M. Glavas, Travis D. Webber, Robert K. Baker, Suheda Erener, Scott D. Covey, Timothy J. Kieffer

**Affiliations:** 1 Department of Cellular and Physiological Sciences, Life Sciences Institute, University of British Columbia, 2350 Health Sciences Mall, Vancouver, BC V6T 1Z3 Canada; 2 Department of Biochemistry and Molecular Biology, University of British Columbia, 2350 Health Sciences Mall, Vancouver, BC V6T 1Z3 Canada; 3 Department of Surgery, University of British Columbia, 2775 Laurel Street, Vancouver, BC V5Z 1M9 Canada; 4 School of Biomedical Engineering, University of British Columbia, 251-2222 Health Sciences Mall, Vancouver, BC V6T 1Z3 Canada

**Keywords:** Homeostasis, Metabolic diseases

## Abstract

Although innate immunity is linked to metabolic health, the effect of leptin signaling in cells from the innate immune system on glucose homeostasis has not been thoroughly investigated. We generated two mouse models using Cre-lox methodology to determine the effect of myeloid cell-specific leptin receptor (*Lepr*) reconstitution and *Lepr* knockdown on in vivo glucose metabolism. Male mice with myeloid cell-specific *Lepr* reconstitution (*Lyz2Cre*^+^*Lepr*^*loxTB/loxTB*^) had better glycemic control as they aged compared to male mice with whole-body transcriptional blockade of *Lepr* (*Lyz2Cre*^*−*^*Lepr*^*loxTB/loxTB*^). In contrast, *Lyz2Cre*^+^*Lepr*^*loxTB/loxTB*^ females only had a trend for diminished hyperglycemia after a prolonged fast. During glucose tolerance tests, *Lyz2Cre*^+^*Lepr*^*loxTB/loxTB*^ males had a mildly improved plasma glucose profile compared to *Cre*^*−*^ controls while *Lyz2Cre*^+^*Lepr*^*loxTB/loxTB*^ females had a similar glucose excursion to their *Cre*^*−*^ controls. Myeloid cell-specific *Lepr* knockdown (*Lyz2Cre*^+^*Lepr*^*flox/flox*^) did not significantly alter body weight, blood glucose, insulin sensitivity, or glucose tolerance in males or females. Expression of the cytokine interleukin 10 (anti-inflammatory) tended to be higher in adipose tissue of male *Lyz2Cre*^+^*Lepr*^*loxTB/loxTB*^ mice (p = 0.0774) while interleukin 6 (pro-inflammatory) was lower in male *Lyz2Cre*^+^*Lepr*^*flox/flox*^ mice (p < 0.05) vs. their respective controls. In conclusion, reconstitution of *Lepr* in cells of myeloid lineage has beneficial effects on glucose metabolism in male mice.

## Introduction

Macrophages can disturb glucose homeostasis and insulin sensitivity by secreting inflammatory cytokines^1,2^. The effect of leptin signaling in cells of myeloid lineage, which include macrophages, on glucose metabolism has not been thoroughly investigated. It has been reported that leptin increases canonical pro-inflammatory cytokine expression, but decreases canonical anti-inflammatory cytokine expression by monocytes/macrophages in vitro in a dose-dependent manner^3–6^. However, leptin has been found to induce a mixed M1 (pro-inflammatory) and M2 (anti-inflammatory) phenotype in monocytes/macrophages^7^ and also to increase anti-inflammatory cytokine expression^8^. The long isoform of the leptin receptor (Leprb) is widely accepted to mediate most of the effects of leptin, including signal transducer and activator of transcription-3 (STAT3) activation, and leptin increases STAT3 activation in murine macrophages^9–14^. Lepr protein is present in bone marrow-derived macrophages from wild-type mice but not in Leprb mutant *db/db* mice^10–12,15^. In macrophages obtained from *db/db* mice, Li et al.^16^ reported that expression of pro-inflammatory and anti-inflammatory cytokines is elevated, but Zykova et al.^17^ did not find differences in expression of pro-inflammatory cytokines in unstimulated conditions. However, characteristics of *db/db* mouse macrophages may be confounded by the metabolic disturbances of the *db/db* model of complete leptin receptor signaling deficiency^18,19^.

Bone marrow transplantation studies involving *db/db* and wild-type mice have yielded conflicting results regarding the effects of leptin signaling in cells of both the innate and adaptive immune systems on cytokine expression and glucose homeostasis^20–23^. Studies using Cre-lox methodology to diminish expression of the leptin receptor in cells of myeloid lineage yielded mild adverse effects on metabolic health, but both sexes were not consistently investigated and glucose metabolism was not always assessed. Mice deficient in all isoforms of the leptin receptor in myeloid cells generated using *Lyz2Cre* gained more weight on standard chow and had increased hepatic lipids, but glucose tolerance was not altered^8^. Any differences between male and female mice were not described. Using *Lyz2Cre* mice and floxed mice that allowed Leprb to be targeted, Scheller et al.^24,25^ also observed a tendency for greater weight gain in male mice, whereas body weight of female Leprb knockdown mice did not differ from controls up to 52 weeks of age. In addition, at 52 weeks of age females had inflammation in the liver. More recently, it was reported that the ability of macrophages from mice with myeloid cell-specific Leprb knockdown (generated using *Lyz2Cre* mice) to eradicate bacterial infection was diminished^26^. Interestingly, mice with myeloid cell-specific Leprb knockdown also had higher circulating levels of leptin vs. Cre^+^ control mice at 8 weeks old, despite similar body weight; male and female mice were matched and pooled in study groups^26^. Moreover, blood glucose concentrations were similar between groups. Lastly, Gao et al.^27^ observed excess weight gain but normal glucose tolerance in male mice with diminished expression of Leprb in cells of myeloid lineage achieved with *Cx3cr1-Cre* mice. The effects in female mice were not reported.

The above investigations of leptin action on cells of myeloid lineage focused on lipid metabolism, bone metabolism, defense against infection, body weight regulation, and neural physiology. Therefore, we performed various experiments to more thoroughly assess in vivo glucose metabolism in mice with diminished expression of Leprb in cells of myeloid lineage. We also generated mice that had *Lepr* expression restored selectively in cells of myeloid lineage and compared their glucose metabolism to that of mice with whole-body transcriptional blockade of *Lepr*. The latter mice have a phenotype similar to *db/db* mice. To the best of our knowledge, a model of myeloid cell-specific *Lepr* reconstitution has not been reported in the literature. Lastly, since leptin is sexually dimorphic, we performed studies in male and female mice and report the results separately.

## Materials and methods

### Study design

We adhered to guidelines of the Canadian Council on Animal Care and the University of British Columbia Animal Care Committee approved all study procedures. Methods are reported according to ARRIVE guidelines. Homozygous *Lyz2Cre*^+^ mice (The Jackson Laboratory, Bar Harbor, ME, USA, Stock #004781) were mated with C57BL/6 mice (The Jackson Laboratory) to give rise to hemizygous *Lyz2Cre*^+^ mice that were subsequently used as breeders in our colonies. All mice used for studies were hemizygous for *Cre*. In the presence of a floxed gene, *Lyz2Cre*^+^ mice have Cre-mediated excision specifically in myeloid cells^28^. *Lyz2Cre* mice are commonly used to knock down a gene in myeloid cells, which include macrophages, via Cre-lox methodology^29–31^. For the *Lepr* knockdown study, mice with myeloid cell-specific *Lepr* knockdown (*Lyz2Cre*^+^*Lepr*^*flox/flox*^) and littermate controls (Flox controls, *Lyz2Cre*^*−*^*Lepr*^*flox/flox*^; Cre controls, *Lyz2Cre*^+^*Lepr*^+*/*+^; wild-type controls, *Lyz2Cre*^*−*^*Lepr*^+*/*+^) were created by starting off mating *Lyz2Cre*^+^ mice with *Lepr*^*flox/flox*^ mice. *Lepr*^*flox/flox*^ mice have *loxP* sites surrounding exon 17 of the leptin receptor gene and Cre-mediated excision causes a frameshift mutation and premature stop codon, thereby impeding expression of the long isoform of the leptin receptor^32–34^. For the *Lepr* reconstitution study, *Lyz2Cre*^+^ mice were mated with *Lepr*^*loxTB/*+^ mice to create mice with myeloid cell-specific *Lepr* reconstitution (*Lyz2Cre*^+^*Lepr*^*loxTB/loxTB*^) and littermate controls, namely mice with global transcriptional blockade of the leptin receptor (*Lyz2Cre*^*−*^*Lepr*^*loxTB/loxTB*^), Cre controls (*Lyz2Cre*^+^*Lepr*^+*/*+^) and wild-type controls (*Lyz2Cre*^*−*^*Lepr*^+*/*+^). We have previously described the *Lepr*^*flox/flox*^ and *Lepr*^*loxTB/*+^ mice used as breeders, including their background^35^. For the *Lepr* knockdown and *Lepr* reconstitution studies, control mice were littermate controls. Ear notches were collected for genotyping and primers used for genotyping are listed in “Supplementary Information S1”. Mice had free access to water and food (Harlan diet #2919 when breeding and Harlan diet #2918 for maintenance) and were kept on a 12 h:12 h light–dark cycle (lights on at 7 a.m.).

### Blood glucose measurements and plasma assays

Body weight was assessed, blood glucose was measured, and blood was collected after fasting for 4 h, starting in the morning, unless stated otherwise. Blood was collected from the saphenous vein. OneTouch Verio glucometers (Life Scan, Burnaby, BC, Canada) were used to measure blood glucose concentrations for the fasting challenge experiments in the *Lepr* reconstitution study. For all other studies, OneTouch Ultra glucometers (Life Scan) were utilized to determine blood glucose concentrations. Values above or below the limits of detection (33.3 and 1.1 mM) were assigned values of 33.3 and 1.1 mM, respectively. Plasma glucose was determined with an in vitro glucose assay that utilizes hexokinase and glucose 6-phosphate dehydrogenase (Sekure Chemistry, Sekisui Diagnostics, Charlottetown, PEI, Canada). Plasma leptin was quantified with a mouse leptin ELISA (Crystal Chem, Downers Grove, IL, USA). A mouse insulin ultrasensitive ELISA (Alpco, Salem, NH, USA) was used for plasma samples from the knockdown study. For the reconstitution study, plasma insulin was measured with the Stellux Chemi Rodent Insulin ELISA (Alpco); plasma samples from *Lyz2Cre*^+^*Lepr*^*loxTB/loxTB*^ and *Lyz2Cre*^*−*^*Lepr*^*loxTB/loxTB*^ mice were diluted 1:3, while plasma samples from *Lyz2Cre*^+^*Lepr*^+*/*+^ and *Lyz2Cre*^*−*^*Lepr*^+*/*+^ mice were run neat. Total and high molecular weight (HMW) adiponectin in plasma was quantified using an ELISA from Alpco. Plasma free fatty acids (FFAs) were quantified as previously described^36^.

### Metabolic tests

For the *Lepr* knockdown study, insulin tolerances tests (ITTs) were performed after a 4 h morning fast in mice 18–21 weeks old. Insulin (Novolin ge Toronto, Novo Nordisk, Mississauga, ON, Canada) was injected i.p. at a dose of 0.75U per kg body weight for males and 0.5U per kg body weight for females. Oral glucose tolerance tests (OGTTs) involved administering 1.5 g glucose per kg body weight, using a 30% glucose solution, to mice 23–27 weeks old after a 4 h morning fast. For fasting challenge experiments, the start of fasting for mice was 5:30–6:30 pm and mice were 38–46 weeks old. Pyruvate tolerance tests (PTTs) were done in mice 42–55 weeks old following an overnight (16 h) fast; sodium pyruvate (Fisher Scientific, Ottawa, ON, Canada) was dissolved in 0.9% NaCl and administered i.p. at a dose of 2 g per kg body weight^37^.

For the *Lepr* reconstitution study, ITTs were performed in mice aged 9–13 weeks old after a 4 h morning fast. For male and female *Cre*^+^*Lepr*^*loxTB/loxTB*^ and *Cre*^*−*^*Lepr*^*loxTB/loxTB*^ mice, the insulin dose was 2U per kg body weight i.p. For male *Lyz2Cre*^+^*Lepr*^+*/*+^ and *Lyz2Cre*^*−*^*Lepr*^+*/*+^ mice, the insulin dose was 0.75U per kg body weight i.p., while female *Lyz2Cre*^+^*Lepr*^+*/*+^ and *Lyz2Cre*^*−*^*Lepr*^+*/*+^ mice were injected with 0.5U per kg body weight i.p. OGTTs were done in mice 25–26 weeks old after an overnight (16 h) fast; all mice received the same dose of glucose (1 g per kg body weight), but while a 50% glucose solution was used for *Cre*^+^*Lepr*^*loxTB/loxTB*^ and *Cre*^*−*^*Lepr*^*loxTB/loxTB*^ mice, a 25% glucose solution was used for *Lyz2Cre*^+^*Lepr*^+*/*+^ and *Lyz2Cre*^*−*^*Lepr*^+*/*+^ mice. Length of fast affects circulating glucose concentrations in obese mice. In order to start the OGTTs when circulating glucose concentrations were normal or near normal in males with *Lepr* reconstitution (*Lyz2Cre*^+^*Lepr*^*loxTB/loxTB*^), OGTTs were performed after an overnight fast. For fasting challenge experiments, fasting started at 5:30–6:30 pm and mice were 19–42 weeks old (equally distributed).

### Assessment of Lepr^flox^ recombination in bone marrow-derived macrophages

Bone marrow from *Lyz2Cre*^+^*Lepr*^*flox/flox*^ and *Lyz2Cre*^*−*^*Lepr*^*flox/flox*^ mice was collected based on the protocol by Pinedo-Torra et al.^38^. Unpolarized macrophages were obtained from bone marrow cells following 7 days of culture in uncharged plastic plates and media consisting of RPMI 1640, 10% FBS, 20% L929 cells conditioned medium, 100 U/ml penicillin/streptomycin, 1X non-essential amino acids, 1 mM sodium pyruvate, and 0.05 mM β-mercaptoethanol^39^. Cells were incubated at 37 °C and 5% CO_2_. DNA was extracted from unpolarized bone marrow-derived macrophages^40^. A qPCR assay was developed to quantify the extent of excision of the floxed *Lepr* allele in DNA isolated from bone marrow-derived macrophages (*Lyz2Cre*^+^*Lepr*^*flox/flox*^ vs. *Lyz2Cre*^*−*^*Lepr*^*flox/flox*^ mice for each sex); the calculation of recombination using this assay as well as the primers and probes used have been described in a previous publication^35^. Briefly, primers were: LeprInt17-F, 5′CCTTTCCAGATAATGCCTGATAGA3′; LeprInt17-R, 5′GCACCACACTTAGCTCCAATA3′; LeprInt16-F, 5′GATCTCACACATACCAGATCC3′; LeprInt16-R, 5′ATTTGATTCCACAAAGTGTTCC3′. Probes were: LeprInt17-FAM, 5′ 56-FAM/TAGGGCGGA/ZEN/TGAACCAGCAAATGT/3IABkFQ; LeprInt16-HEX probe, 5′/5HEX/AGGAACTTCG/ZEN/GAATAGGAACTTCGAATTCCTCGAGATC/3IABkFQ.

### RT-qPCR

The RNeasy lipid tissue mini kit (Qiagen, Hilden, Germany) was used to extract RNA from perigonadal white adipose tissue samples of males and cDNA was subsequently obtained, as previously described^35^. Primer sequences: Arginase 1 (*Arg1*), Forward-5′TCTACATCACAGAAGAAATTTACAAGA3′ and Reverse-5′TTAGGTGGTTTAAGGTAGTCAGTCC3′; Interleukin 6 (*Il6*), Forward-5′CCAATTTCCAATGCTCTCCT3′ and Reverse-5′ACCACAGTGAGGAATGTCCA3′; Interleukin 10 (*Il10*), Forward-5′GGAGCAGGTGAAGAGTGATTTTAA3′ and Reverse-5′TGCAGGTGTTTTAGCTTTTCATTT3′; Tumor necrosis α (*Tnf*), Forward-5′ACGGCATGGATCTCAAAGAC3′ and Reverse-5′AGATAGCAAATCGGCTGACG3′. The reference transcript was peptidylpropyl isomerase A (*Ppia*); primer sequences were Forward-5′AGCTCTGAGCACTGGAGAGA3′ and Reverse-5′GCCAGGACCTGTATGCTTTA3′. For samples without amplification, a Cq value of 39 was assigned. Calculation of relative expression (to wild-type controls for the *Lepr* reconstitution study and Flox controls for the *Lepr* knockdown study) was done with the Pfaffl equation.

### Statistical analyses

Results are displayed as mean ± SEM or box and whisker plots with individual points. Box and whisker plots have lines representing the median, 25th percentile, and 75th percentile, and whiskers representing the highest and lowest values. Statistical analyses were done using GraphPad Prism 8 and consisted of: (1) unpaired t-test when comparing two genotypes; (2) one-way ANOVA with Tukey’s post-hoc test when comparing more than two genotypes, except for analyses of RT-qPCR results where Dunnett’s post-hoc test was used due to small sample sizes; or (3) for parameters that changed over time, repeated measures two-way ANOVA. For the latter, when statistical significance was obtained for the main effect of genotype and/or interaction, comparison of genotypes was done at each time point using a post-hoc analysis, namely Bonferroni for two genotypes and Tukey for more than two genotypes. Analysis of plasma insulin concentrations during the OGTT in the *Lepr* reconstitution study was done with a mixed-effects model because: (1) values were missing at some timepoints for some mice due to difficulty in obtaining sufficient plasma from *Cre*^+^*Lepr*^*loxTB/loxTB*^ and *Cre*^*−*^*Lepr*^*loxTB/loxTB*^ mice and (2) for 1 *Cre*^+^*Lepr*^*loxTB/loxTB*^ male, 1 *Cre*^+^*Lepr*^*loxTB/loxTB*^ female, and 1 *Cre*^*−*^*Lepr*^+*/*+^ female, insulin ELISA results fell outside the standard curve. Tukey’s post-hoc test was used when statistical significance was obtained for the main effect of genotype and/or interaction with the mixed-effects model. α = 0.05.

## Results

**Figure 1:**
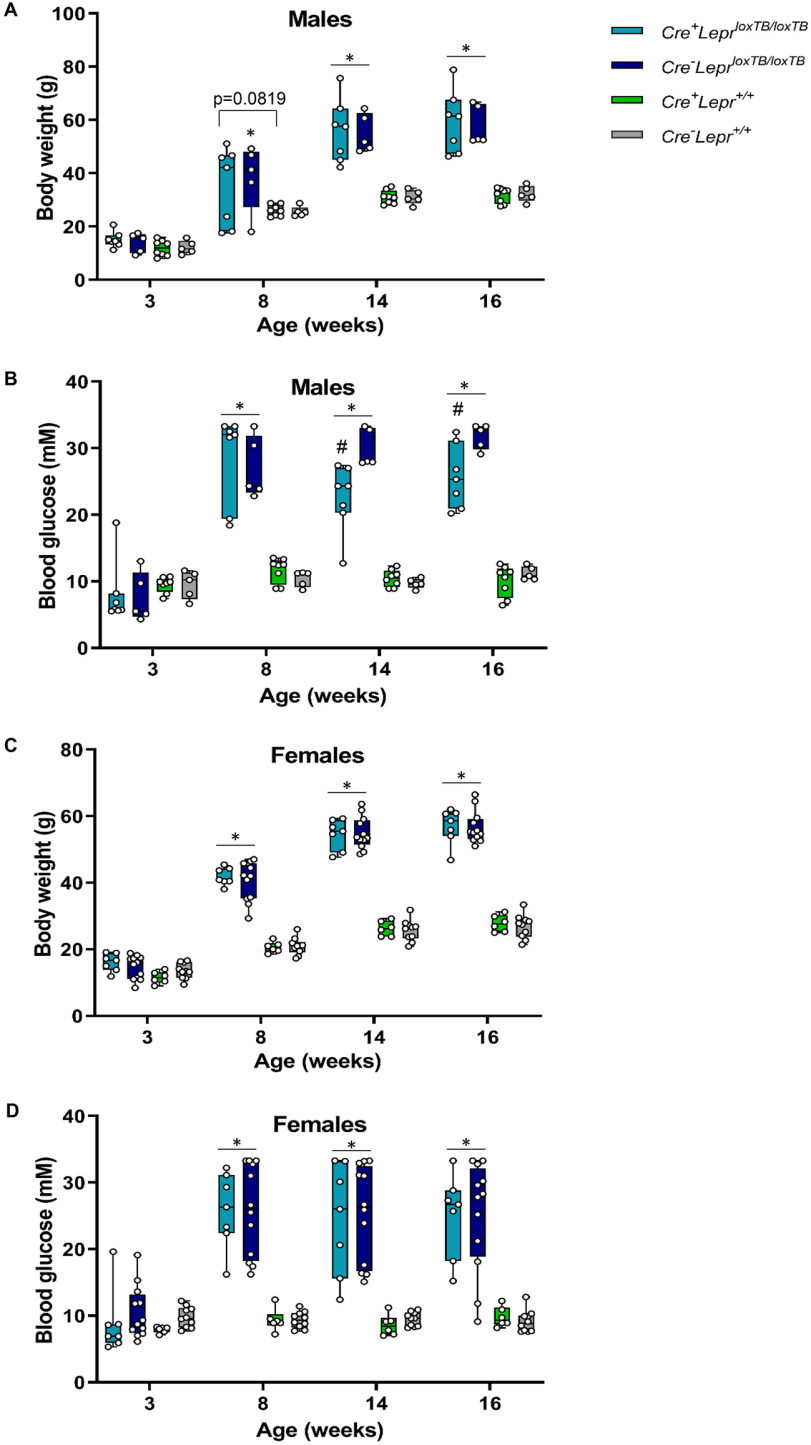
Body weight (**A**, **C**) and blood glucose (**B**, **D**) of mice from the *Lepr* reconstitution study at different ages. Parameters obtained after a 4 h fast. In (**A**) and (**B**), n = 7 for *Cre*^+^*Lepr*^*loxTB/loxTB*^, n = 5 for *Cre*^*−*^*Lepr*^*loxTB/loxTB*^, n = 8 for *Cre*^+^*Lepr*^+*/*+^, and n = 5 for *Cre*^*−*^*Lepr*^+*/*+^. In (**C**) and (**D**), n = 7 for *Cre*^+^*Lepr*^*loxTB/loxTB*^, n = 12 for *Cre*^*−*^*Lepr*^*loxTB/loxTB*^, n = 6 for *Cre*^+^*Lepr*^+*/*+^, and n = 10 for *Cre*^*−*^*Lepr*^+*/*+^. Performed repeated measures two-way ANOVA with post-hoc analysis. *p < 0.05, *Cre*^+^*Lepr*^*loxTB/loxTB*^ and *Cre*^*−*^* Lepr*^*loxTB/loxTB*^ vs. *Cre*^+^*Lepr*^+*/*+^ and *Cre*^*−*^*Lepr*^+*/*+^ (except in A at 8 weeks old, where *p < 0.05, *Cre*^*−*^* Lepr*^*loxTB/loxTB*^ vs. *Cre*^+^*Lepr*^+*/*+^ and *Cre*^*−*^*Lepr*^+*/*+^); ^#^p < 0.05 vs. *Cre*^*−*^*Lepr*^*loxTB/loxTB*^. Main effect of time (p < 0.05) in (**A**–**D**).

We investigated whether reconstituting *Lepr* selectively in myeloid cells of mice with whole-body *Lepr* transcriptional blockade, which are characterized by obesity, hyperinsulinemia, and hyperleptinemia^35^, would alter glucose metabolism. Genotyping results from ear notch samples are shown in Supplementary Fig. S1. Among males, mice with myeloid cell-specific *Lepr* reconstitution (*Cre*^+^*Lepr*^*loxTB/loxTB*^) and mice with global transcriptional blockade of *Lepr* (*Cre*^*−*^*Lepr*^*loxTB/loxTB*^) had similar body weight to Cre controls (*Cre*^+^*Lep*^+*/*+^) and wild-type controls (*Cre*^*−*^*Lep*^+*/*+^) at 3 weeks old (Fig. 1A). At 14 and 16 weeks old, male mice with myeloid cell-specific *Lepr* reconstitution and whole-body transcriptional blockade of *Lepr* were obese to a similar extent. Blood glucose was not significantly different at 3 weeks old between the 4 genotypes, but by 8 weeks of age, male mice with global transcriptional blockade of *Lepr* and myeloid cell-specific *Lepr* reconstitution were hyperglycemic (p < 0.05; Fig. 1B). Interestingly, at 14 and 16 weeks old, male mice with myeloid cell-specific *Lepr* reconstitution had reduced hyperglycemia compared to mice with global transcriptional blockade of *Lepr* (p < 0.05). Among females, body weight and blood glucose were comparable at 3 weeks old between the four genotypes, but at 8, 14, and 16 weeks old, mice with myeloid cell-specific *Lepr* reconstitution and mice with global transcriptional blockade of *Lepr* were similarly obese and hyperglycemic (p < 0.05 vs. Cre controls and wild-type controls; Fig. 1C,D).

**Figure 2:**
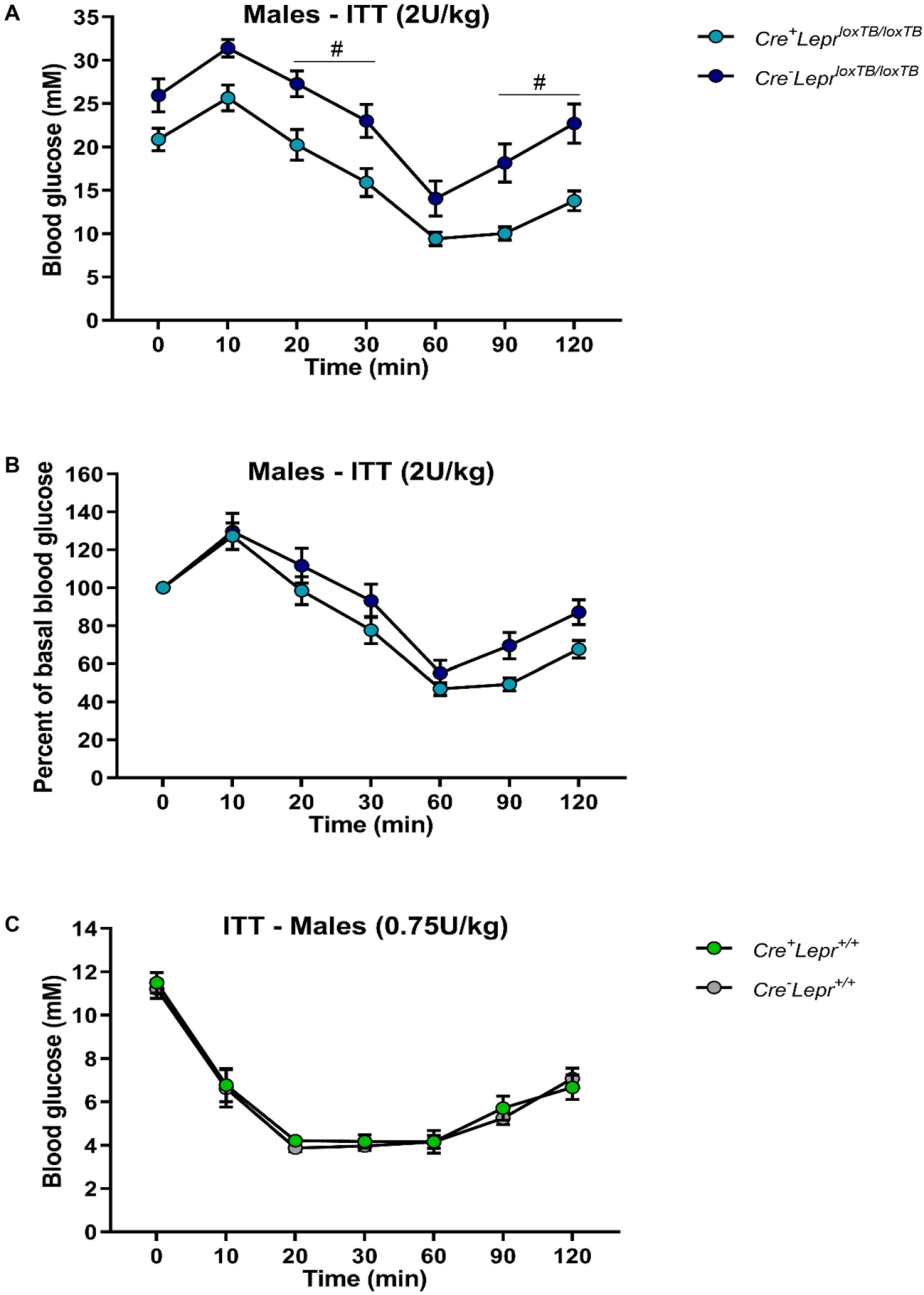
Insulin tolerance tests (ITTs) in male mice from the *Lepr* reconstitution study. In (**A**), the insulin dose was 2U per kg of body weight. In (**B**), blood glucose concentration of each mouse in (**A**) was normalized to its basal (0 min) blood glucose concentration. In (**C**), the insulin dose was 0.75 U per kg of body weight. In (**A**) and (**B**), n = 18 for *Cre*^+^*Lepr*^*loxTB/loxTB*^ and n = 14 for *Cre*^*−*^*Lepr*^*loxTB/loxTB*^; in (**C**), n = 15 for *Cre*^+^*Lepr*^+*/*+^ and n = 11 for *Cre*^*−*^*Lepr*^+*/*+^. Performed repeated measures two-way ANOVA with post-hoc analysis. ^#^p < 0.05 vs. *Cre*^*−*^*Lepr*^*loxTB/loxTB*^. Main effect of time (p < 0.05) in (**A**–**C**).

**Figure 3:**
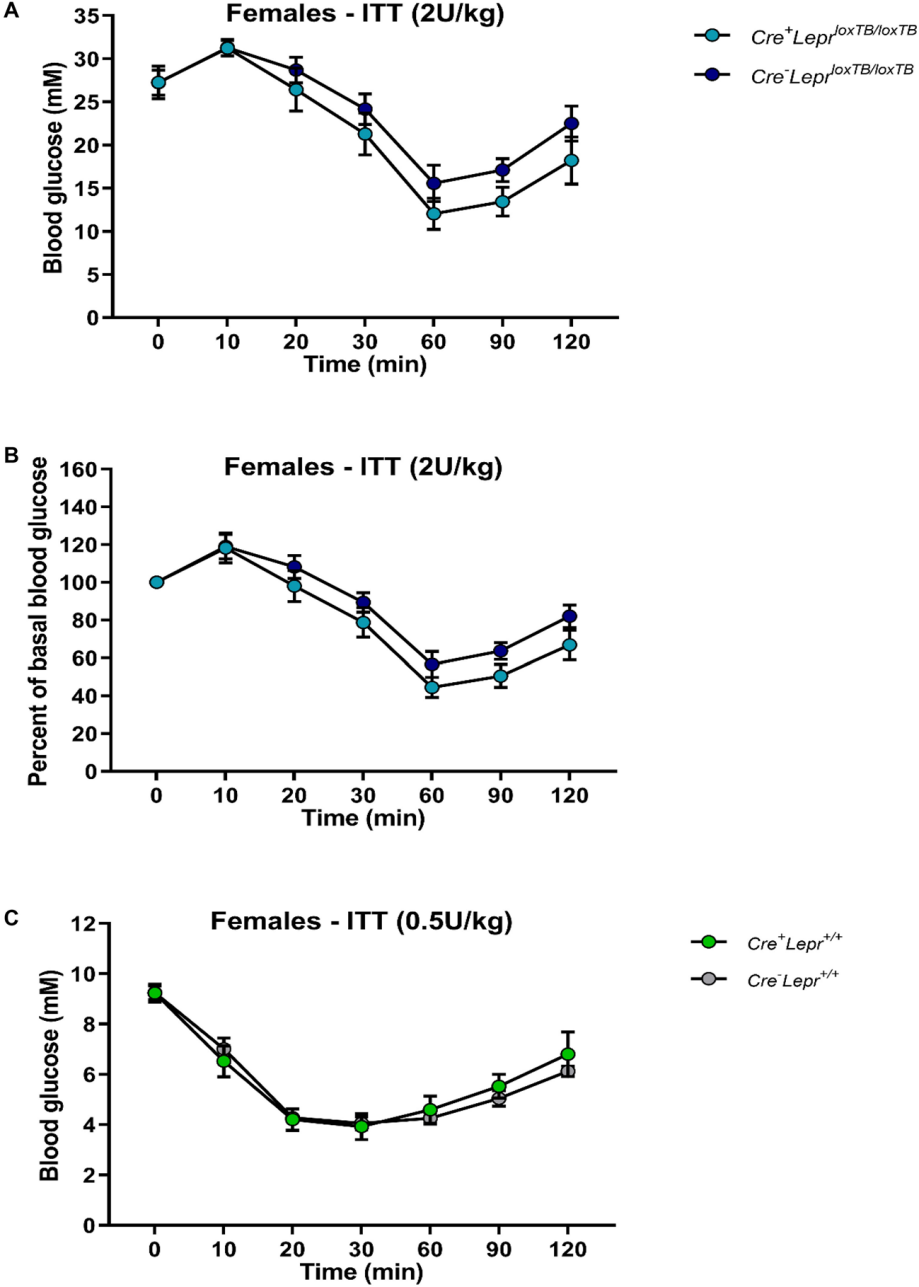
Insulin tolerance tests (ITTs) in female mice from the *Lepr* reconstitution study. In (**A**), the insulin dose was 2U per kg of body weight. In (**B**), blood glucose concentration of each mouse in (**A**) was normalized to its basal (0 min) blood glucose concentration. In (**C**), the insulin dose was 0.5 U per kg of body weight. In (**A**) and (**B**), n = 8 for *Cre*^+^*Lepr*^*loxTB/loxTB*^ and n = 15 for *Cre*^*−*^*Lepr*^*loxTB/loxTB*^; in (**C**), n = 10 for *Cre*^+^*Lepr*^+*/*+^ and n = 11 for *Cre*^*−*^*Lepr*^+*/*+^. Performed repeated measures two-way ANOVA with post-hoc analysis. Main effect of time (p < 0.05) in (**A**–**C**).

Male mice with myeloid cell-specific *Lepr* reconstitution, aged 9–13 weeks old, had significantly lower blood glucose relative to *Cre*^*−*^*Lepr*^*loxTB/loxTB*^ mice at 20, 30, 90, and 120 min of the ITT (p < 0.05; Fig. 2A), but there were no statistically significant differences between these two genotypes during the ITT when blood glucose concentrations were expressed as a percentage of basal (0 min) blood glucose concentrations (Fig. 2B). Male Cre controls and male wild-type controls had a similar blood glucose response during the ITT (Fig. 2C). Among females, the blood glucose profile during the ITT was similar in obese mice (Fig. 3A,B) as well as lean controls (Fig. 3C).

**Figure 4:**
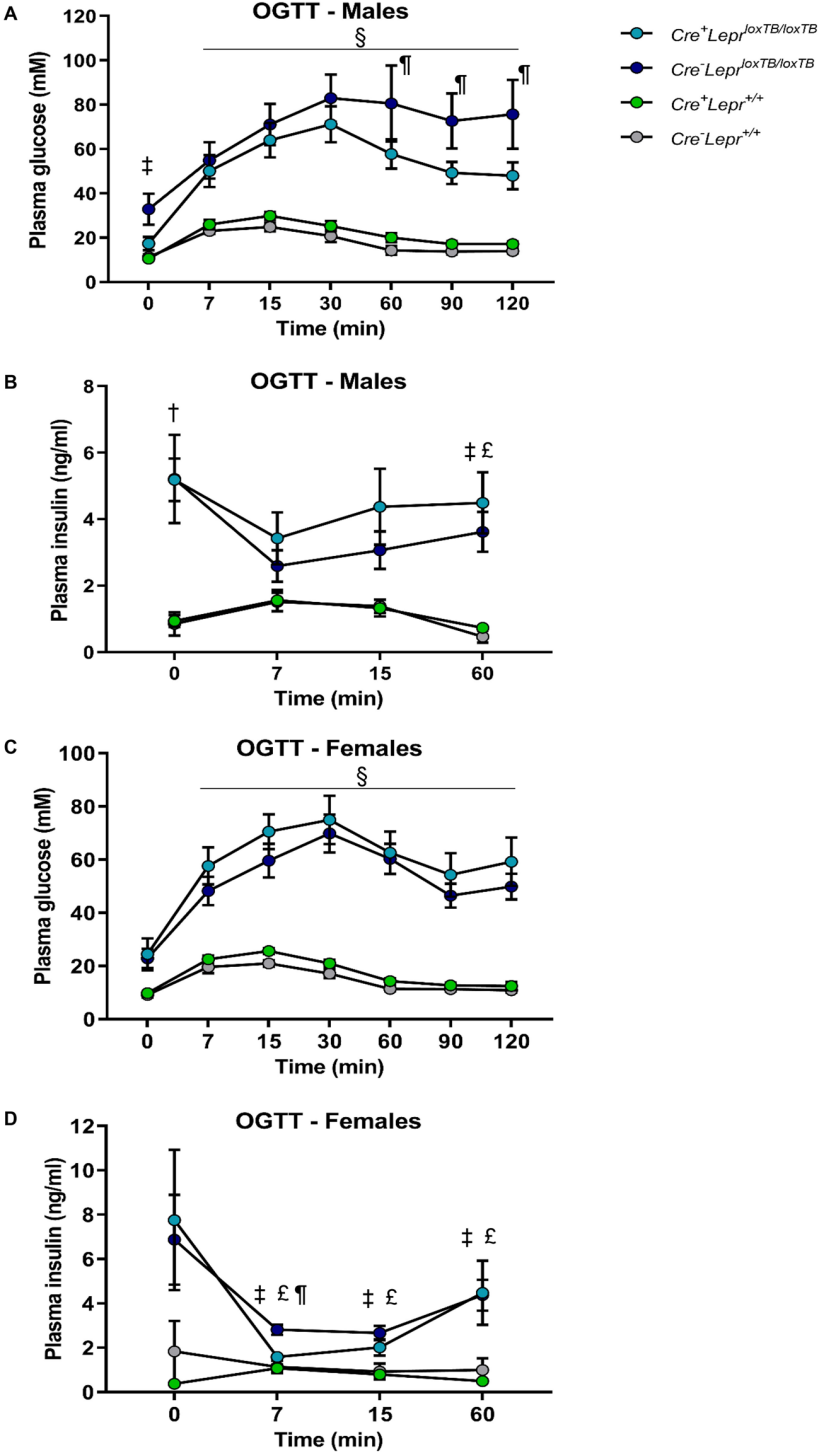
Plasma glucose (**A**, **C**) and plasma insulin (**B**, **D**) during oral glucose tolerance tests (OGTTs) in mice from the *Lepr* reconstitution study. In (**A**), n = 6 for *Cre*^+^*Lepr*^*loxTB/loxTB*^, n = 5 for *Cre*^*−*^*Lepr*^*loxTB/loxTB*^, n = 9 for *Cre*^+^*Lepr*^+*/*+^, and n = 6 for *Cre*^*−*^*Lepr*^+*/*+^. In (**B**), for *Cre*^+^*Lepr*^*loxTB/loxTB*^, n = 5 at all timepoints, except at t = 60 min, where n = 3; for *Cre*^*−*^*Lepr*^*loxTB/loxTB*^, n = 5 at all timepoints, except at t = 7 min and t = 15 min, where n = 4; for *Cre*^+^*Lepr*^+*/*+^, n = 9 at all timepoints; for *Cre*^*−*^*Lepr*^+*/*+^, n = 5 at all timepoints. In (**C**), n = 6 for *Cre*^+^*Lepr*^*loxTB/loxTB*^, n = 9 for *Cre*^*−*^*Lepr*^*loxTB/loxTB*^, n = 8 for *Cre*^+^*Lepr*^+*/*+^, and n = 9 for *Cre*^*−*^*Lepr*^+*/*+^. In (**D**), for *Cre*^+^*Lepr*^*loxTB/loxTB*^, n = 6 at all timepoints, except at t = 0 min, where n = 5, at t = 7 min, where n = 4, and t = 15 min, where n = 5; for *Cre*^*−*^*Lepr*^*loxTB/loxTB*^, n = 8 at all timepoints, except at t = 7 min, where n = 6, t = 15 min, where n = 7, and t = 60 min, where n = 7; for *Cre*^+^*Lepr*^+*/*+^, n = 8 at all timepoints, except at t = 0 min, where n = 7; for *Cre*^*−*^*Lepr*^+*/*+^, n = 6 at all timepoints, except at t = 0 min, where n = 3, and t = 7 min, where n = 5. In (**A**) and (**C**), repeated measures two-way ANOVA with post-hoc analysis was performed. In (**B**) and (**D**), a mixed-effects model with post-hoc analysis was carried out. ^§^p < 0.05, *Cre*^+^*Lepr*^*loxTB/loxTB*^ and *Cre*^*−*^* Lepr*^*loxTB/loxTB*^ vs. *Cre*^+^*Lepr*^+*/*+^ and *Cre*^*−*^*Lepr*^+*/*+^; ^†^p < 0.05, *Cre*^+^*Lepr*^*loxTB/loxTB*^ vs. *Cre*^+^*Lepr*^+*/*+^ and *Cre*^*−*^*Lepr*^+*/*+^; ^‡^p < 0.05, *Cre*^*−*^*Lepr*^*loxTB/loxTB*^ vs. *Cre*^+^*Lepr*^+*/*+^; ^£^p < 0.05, *Cre*^*−*^*Lepr*^*loxTB/loxTB*^ vs. *Cre*^*−*^*Lepr*^+*/*+^; ^&^p < 0.05, *Cre*^+^*Lepr*^*loxTB/loxTB*^ vs. *Cre*^+^*Lepr*^+*/*+^; ^¶^p < 0.05 *Cre*^+^*Lepr*^*loxTB/loxTB*^ vs. *Cre*^*−*^*Lepr*^*loxTB/loxTB*^. Main effect of time (p < 0.05) in (**A**, **C**, **D**).

Male obese mice with and without *Lepr* reconstitution had impaired glucose tolerance during the OGTT at 25–26 weeks of age (p < 0.05; Fig. 4A), but male mice with myeloid cell-specific *Lepr* reconstitution had moderately improved glucose tolerance compared to male mice with global transcriptional blockade of *Lepr* (p < 0.05 at 60, 90, and 120 min; Fig. 4A). During the OGTT, male obese mice with and without *Lepr* reconstitution were hyperinsulinemic compared to Cre controls and wild-type controls (Fig. 4B). We have previously observed that *Cre*^*−*^*Lepr*^*loxTB/loxTB*^ mice have a reduction in plasma insulin concentrations following an oral glucose challenge^35^. Female mice with *Lepr* reconstitution and global transcriptional blockade of *Lepr* were similarly glucose intolerant vs. Cre controls and wild-type controls (p < 0.05; Fig. 4C). Female obese mice with and without *Lepr* reconstitution were hyperinsulinemic during the OGTT, but the female mice with *Lepr* reconstitution were less so (Fig. 4D).

**Figure 5:**
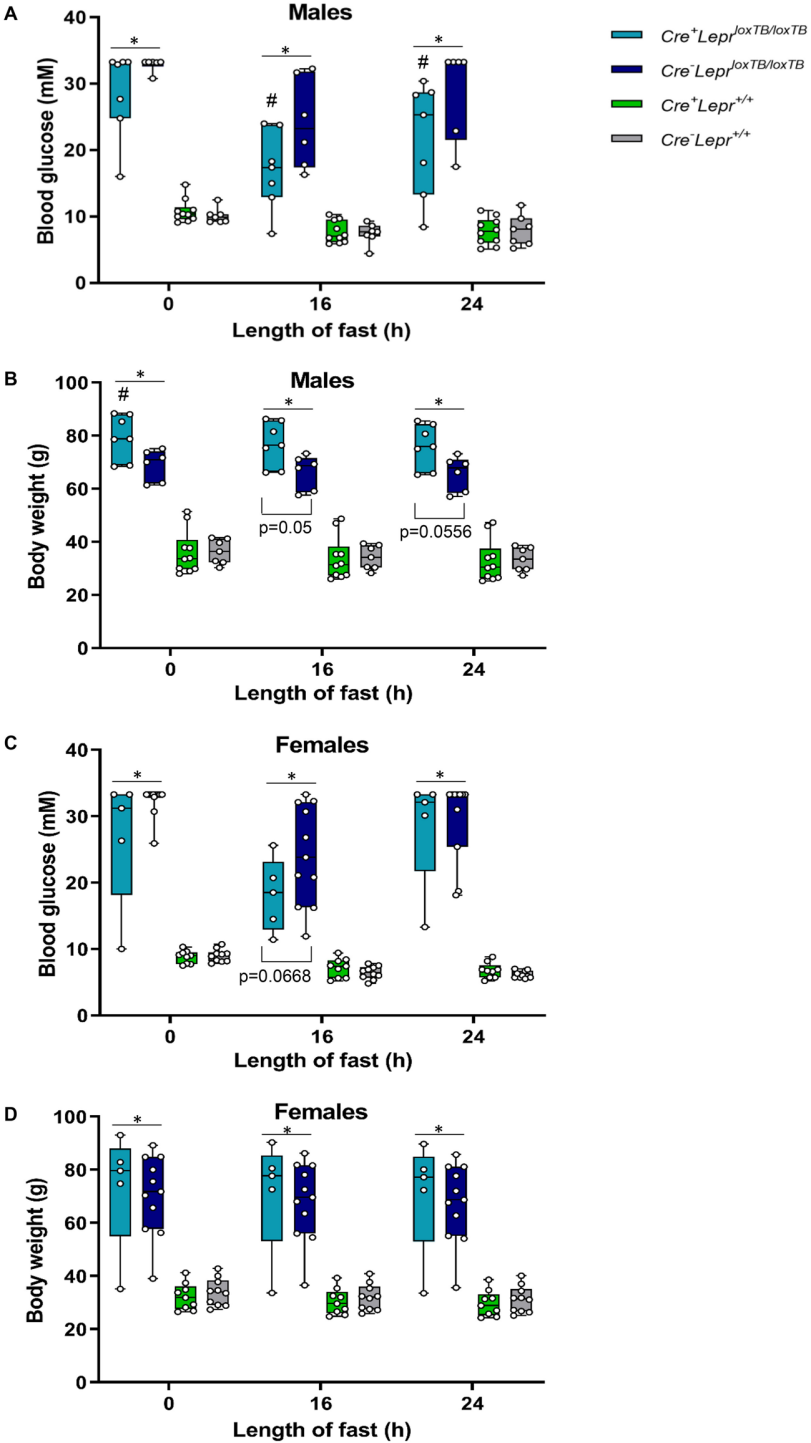
Blood glucose concentration (**A**, **C**) and body weight (**B**, **D**) of mice from the *Lepr* reconstitution study at different fasting times. In (**A**) and (**B**), n = 7 for *Cre*^+^*Lepr*^*loxTB/loxTB*^, n = 6 for *Cre*^*−*^*Lepr*^*loxTB/loxTB*^, n = 10 for *Cre*^+^*Lepr*^+*/*+^, and n = 7 for *Cre*^*−*^*Lepr*^+*/*+^. In (**C**) and (**D**), n = 5 for *Cre*^+^*Lepr*^*loxTB/loxTB*^, n = 11 for *Cre*^*−*^*Lepr*^*loxTB/loxTB*^, n = 9 for *Cre*^+^*Lepr*^+*/*+^, and n = 10 for *Cre*^*−*^*Lepr*^+*/*+^. Performed repeated measures two-way ANOVA with post-hoc analysis. *p < 0.05, *Cre*^+^*Lepr*^*loxTB/loxTB*^ and *Cre*^*−*^* Lepr*^*loxTB/loxTB*^ vs. *Cre*^+^*Lepr*^+*/*+^ and *Cre*^*−*^*Lepr*^+*/*+^; ^#^p < 0.05 vs. *Cre*^*−*^*Lepr*^*loxTB/loxTB*^. Main effect of time (p < 0.05) in (**A**–**D**).

Blood glucose was also determined after different fasting durations in older age mice. In males, hyperglycemia was lower in mice with myeloid cell-specific *Lepr* reconstitution vs. mice with global transcriptional blockade of *Lepr* at 0 h, 16 h, and 24 h of fasting (p < 0.05; Fig. 5A). In females, there was a trend (p = 0.0668) towards improved glycemia after 16 h of fasting in mice with myeloid cell-specific *Lepr* reconstitution vs. mice with whole-body transcriptional blockade of *Lepr* (Fig. 5C). Differences in body weight between mice with myeloid cell-specific *Lepr* reconstitution and global transcriptional blockade of *Lepr* compared to Cre controls and wild-type controls were retained throughout fasting (p < 0.05; Fig. 5B,D). Interestingly, male mice with global transcriptional blockade of *Lepr* were lighter than male mice with myeloid cell-specific *Lepr* reconstitution (Fig. 5B; p < 0.05), which suggests that the former had poorer chronic glycemic control resulting in weight loss at an older age.

**Figure 6:**
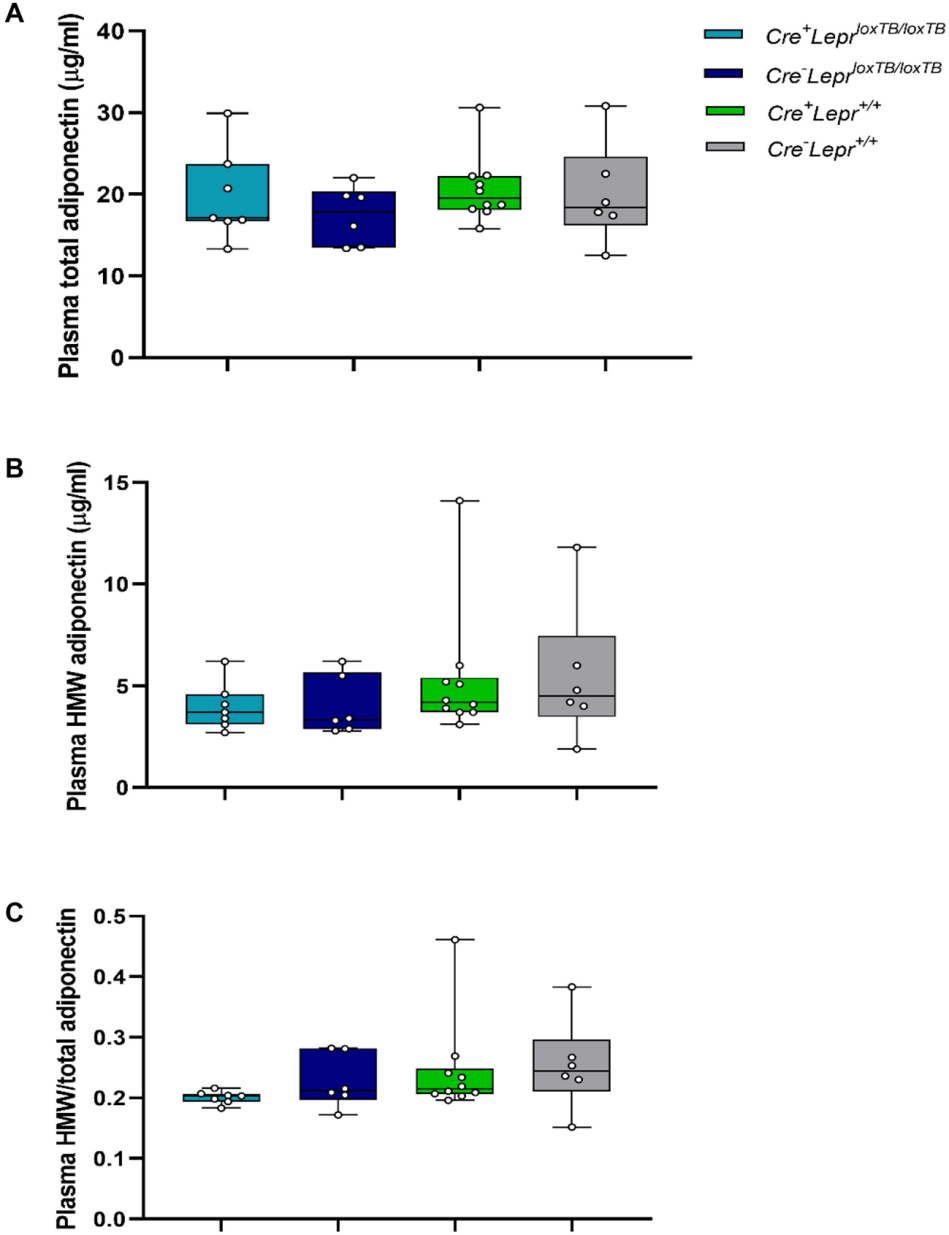
Concentration of total adiponectin (**A**), concentration of high molecular weight (HMW) adiponectin (**B**), and ratio of HMW to total adiponectin (**C**) in plasma obtained from male mice in the *Lepr* reconstitution study after a 16 h fast. In (**A**–**C**), n = 7 for *Cre*^+^*Lepr*^*loxTB/loxTB*^, n = 6 for *Cre*^*−*^*Lepr*^*loxTB/loxTB*^, n = 10 for *Cre*^+^*Lepr*^+*/*+^, and n = 6 for *Cre*^*−*^*Lepr*^+*/*+^. Performed one-way ANOVA with Tukey’s post-hoc test.

We also tested if circulating levels of the adipokine adiponectin differed between genotypes after a prolonged fast. Some bone marrow transplantation studies have reported improved adiponectin levels in mice with intact leptin signaling only in immune cells^20,21^. Adiponectin can reduce hepatic glucose production and expression of gluconeogenic genes independently of insulin sensitivity via AMP-activated protein kinase (AMPK)^41^ and the decrease in blood glucose concentration in males with *Lepr* reconstitution after a prolonged fast suggests that these mice have reduced hepatic glucose production. We found that plasma adiponectin levels were not significantly different among male mice in the *Lepr* reconstitution study after a 16 h fast (Fig. 6).

**Figure 7:**
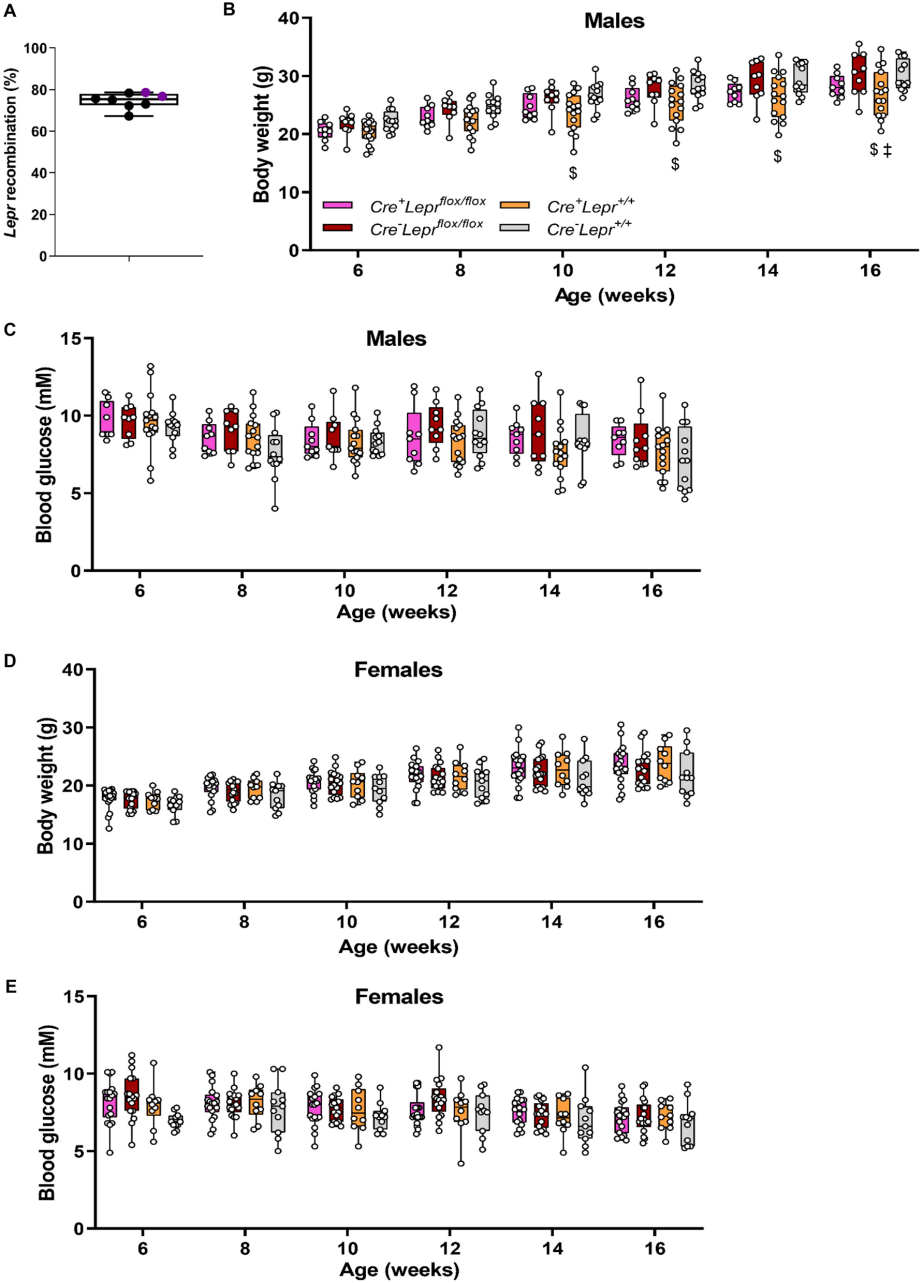
Recombination in bone marrow-derived macrophages of mice from the *Lepr* knockdown study; male mice are represented by black circles and female mice are represented by purple circles (**A**). Body weight (**B**, **D**) and blood glucose (**C**, **E**) of mice from the *Lepr* knockdown study at different ages; parameters obtained after a 4 h fast. In (**B**) and (**C**), n = 9 for *Cre*^+^*Lepr*^*flox/flox*^, n = 9 for *Cre*^*−*^*Lepr*^*flox/flox*^, n = 15 for *Cre*^+^*Lepr*^+*/*+^, and n = 12 for *Cre*^*−*^*Lepr*^+*/*+^. In (**D**) and (**E**), n = 18 for *Cre*^+^*Lepr*^*flox/flox*^, n = 17 for *Cre*^*−*^*Lepr*^*flox/flox*^, n = 10 for *Cre*^+^*Lepr*^+*/*+^, and n = 11 for *Cre*^*−*^*Lepr*^+*/*+^. Performed repeated measures two-way ANOVA with post-hoc analysis in (**B**–**E**). ^$^p < 0.05, *Cre*^+^*Lepr*^+*/*+^ vs. *Cre*^*−*^*Lepr*^+*/*+^; ^‡^p < 0.05, *Cre*^*−*^*Lepr*^*flox/flox*^ vs. *Cre*^+^*Lepr*^+*/*+^. Main effect of time (p < 0.05) in (**B**–**E**).

**Figure 8:**
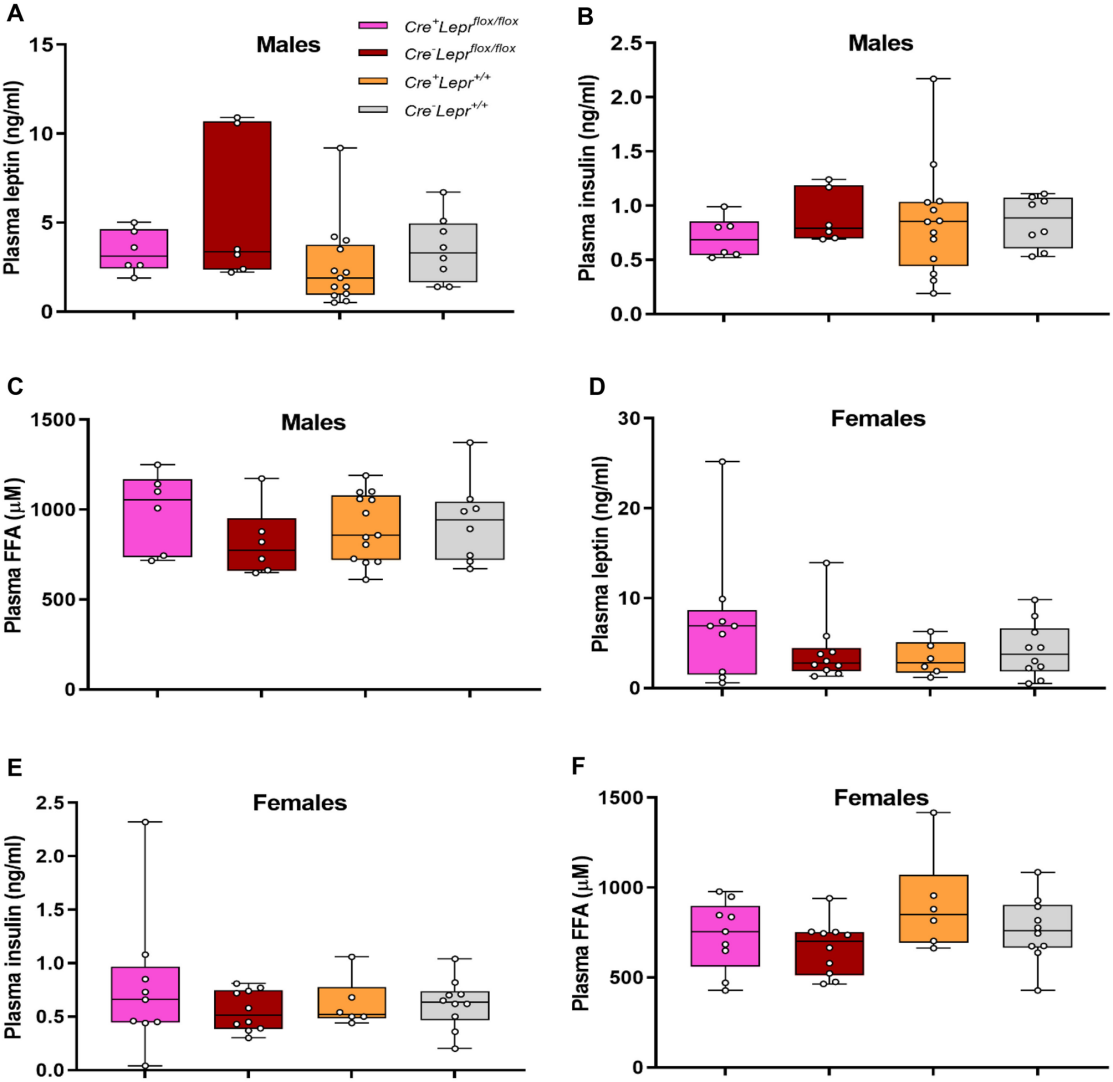
Plasma leptin (**A**, **D**), insulin (**B**, **E**), and free fatty acids (FFA) (**C**, **F**) of 12 week old mice from the *Lepr* knockdown study. Parameters obtained after 4 h fast. For males, n = 6 for *Cre*^+^*Lepr*^*flox/flox*^, n = 6 for *Cre*^*−*^*Lepr*^*flox/flox*^, n = 13 for *Cre*^+^*Lepr*^+*/*+^, and n = 8 for *Cre*^*−*^*Lepr*^+*/*+^. For females, n = 9 for *Cre*^+^*Lepr*^*flox/flox*^, n = 10 for *Cre*^*−*^*Lepr*^*flox/flox*^, n = 6 for *Cre*^+^*Lepr*^+*/*+^, and n = 10 for *Cre*^*−*^*Lepr*^+*/*+^. Performed one-way ANOVA with Tukey’s post-hoc test.

**Figure 9:**
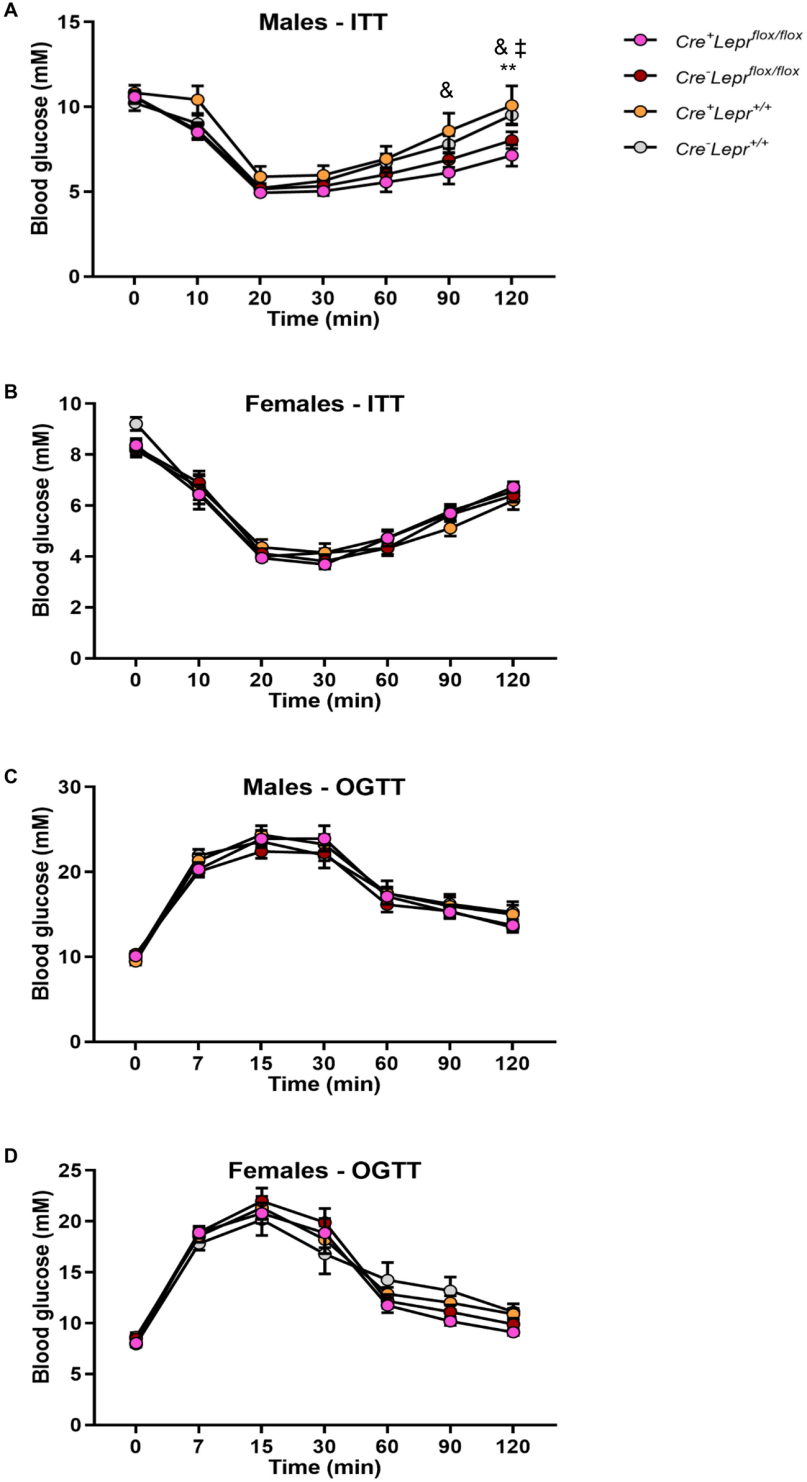
Insulin tolerance tests (ITTs) (**A**, **B**) and oral glucose tolerance tests (OGTTs) (**C**, **D**) in mice from the *Lepr* knockdown study. In (**A**), n = 18 for *Cre*^+^*Lepr*^*flox/flox*^, n = 20 for *Cre*^*−*^*Lepr*^*flox/flox*^, n = 12 for *Cre*^+^*Lepr*^+*/*+^, and n = 13 for *Cre*^*−*^*Lepr*^+*/*+^. In (**B**), n = 20 for *Cre*^+^*Lepr*^*flox/flox*^, n = 17 for *Cre*^*−*^*Lepr*^*flox/flox*^, n = 9 for *Cre*^+^*Lepr*^+*/*+^, and n = 8 for *Cre*^*−*^*Lepr*^+*/*+^. In (**C**), n = 15 for *Cre*^+^*Lepr*^*flox/flox*^, n = 18 for *Cre*^*−*^*Lepr*^*flox/flox*^, n = 11 for *Cre*^+^*Lepr*^+*/*+^, and n = 12 for *Cre*^*−*^*Lepr*^+*/*+^. In (**D**), n = 20 for *Cre*^+^*Lepr*^*flox/flox*^, n = 16 for *Cre*^*−*^*Lepr*^*flox/flox*^, n = 9 for *Cre*^+^*Lepr*^+*/*+^, and n = 8 for *Cre*^*−*^*Lepr*^+*/*+^. Performed repeated measures two-way ANOVA with post-hoc analysis. ^‡^p < 0.05, *Cre*^*−*^*Lepr*^*flox/flox*^ vs. *Cre*^+^*Lepr*^+*/*+^; ^&^p < 0.05, *Cre*^+^*Lepr*^*flox/flox*^ vs. *Cre*^+^*Lepr*^+*/*+^; **p < 0.05, *Cre*^+^*Lepr*^*flox/flox*^ vs. *Cre*^*−*^*Lepr*^+*/*+^. Main effect of time (p < 0.05) in (**A**–**D**).

**Figure 10:**
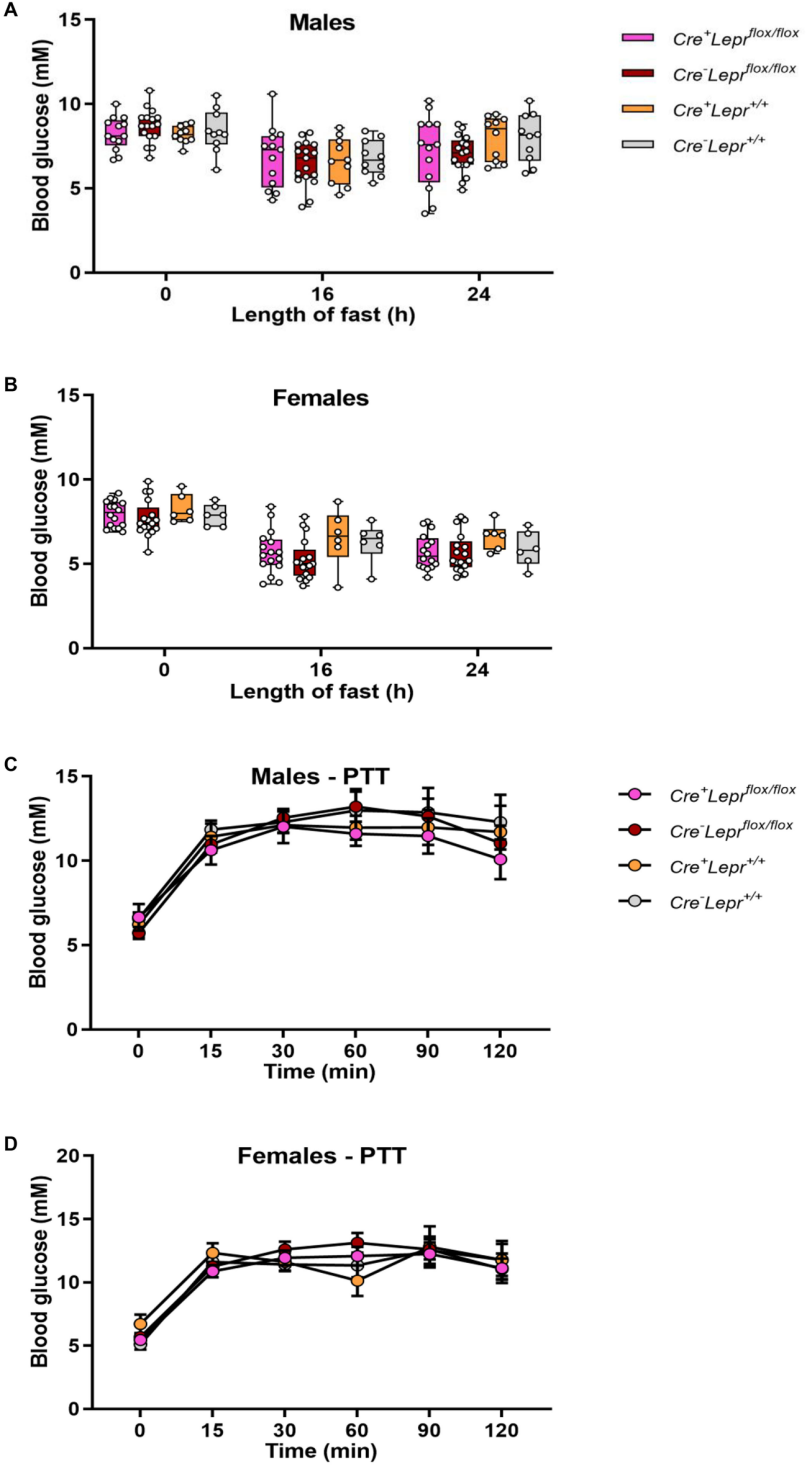
Blood glucose at different fasting times (**A**, **B**) and pyruvate tolerance tests (PTTs) (**C**, **D**) in mice from the *Lepr* knockdown study. In (**A**), n = 13 for *Cre*^+^*Lepr*^*flox/flox*^, n = 17 for *Cre*^*−*^*Lepr*^*flox/flox*^, n = 10 for *Cre*^+^*Lepr*^+*/*+^, and n = 10 for *Cre*^*−*^*Lepr*^+*/*+^. In (**B**), n = 16 for *Cre*^+^*Lepr*^*flox/flox*^, n = 17 for *Cre*^*−*^*Lepr*^*flox/flox*^, n = 6 for *Cre*^+^*Lepr*^+*/*+^, and n = 6 for *Cre*^*−*^*Lepr*^+*/*+^. In (**C**), n = 13 for *Cre*^+^*Lepr*^*flox/flox*^, n = 17 for *Cre*^*−*^*Lepr*^*flox/flox*^, n = 8 for *Cre*^+^*Lepr*^+*/*+^, and n = 9 for *Cre*^*−*^*Lepr*^+*/*+^. In (**D**), n = 16 for *Cre*^+^*Lepr*^*flox/flox*^, n = 17 for *Cre*^*−*^*Lepr*^*flox/flox*^, n = 5 for *Cre*^+^*Lepr*^+*/*+^, and n = 6 for *Cre*^*−*^*Lepr*^+*/*+^. Performed repeated measures two-way ANOVA with post-hoc analysis. Main effect of time (p < 0.05) in (**A**–**D**).

Recombination in bone marrow-derived macrophages from *Lyz2Cre*^+^*Lepr*^*flox/flox*^ mice was substantial, approximately 75% (Fig. 7A). Genotyping results from ear notch samples are in Supplementary Fig. S2. Among males and females, there were no statistically significant differences in body weight, blood glucose, and plasma leptin, insulin and FFAs between mice with myeloid cell-specific *Lepr* knockdown and controls (Figs. 7B–E and 8). There were also no statistically significant differences during the ITT between mice with *Lepr* knockdown and controls (Fig. 9A,B). Results for the OGTT and blood glucose at different fasting times were similar between mice with *Lepr* knockdown and controls (Figs. 9C,D and 10A,B). A PTT was performed after an overnight fast to assess gluconeogenic flux. Blood glucose concentrations were similar between genotypes throughout the PTT in males and females (Fig. 10C,D). Hence, we did not detect any alterations in the phenotype of mice with myeloid-cell specific *Lepr* knockdown, which were characterized by normal weight as well as normal levels of circulating insulin and leptin.

**Figure 11:**
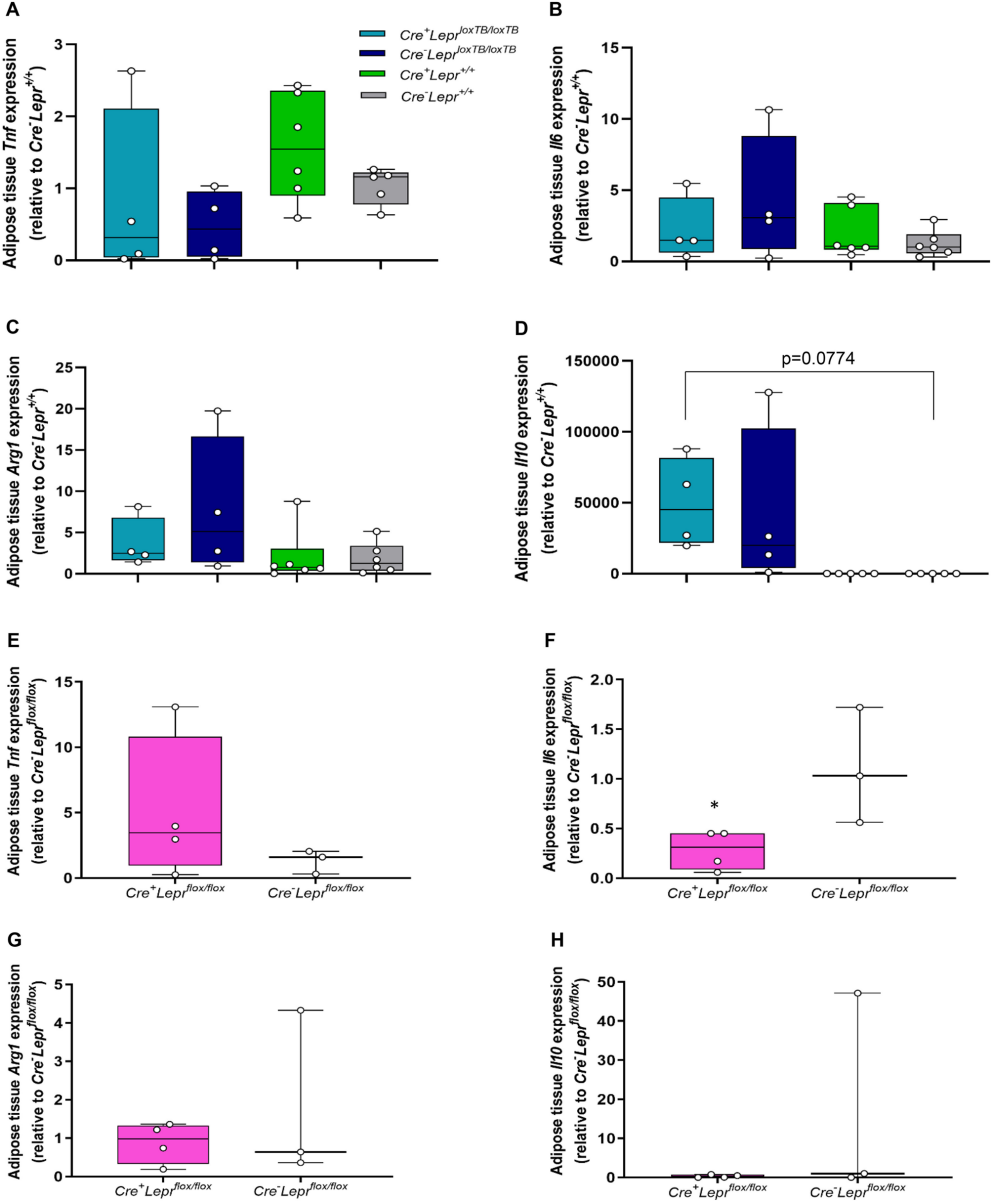
Adipose tissue pro- and anti-inflammatory cytokine expression assessed by RT-qPCR in males from the *Lepr* reconstitution and *Lepr* knockdown studies. In (**A**), n = 4 for *Cre*^+^*Lepr*^*loxTB/loxTB*^, n = 4 for *Cre*^*−*^*Lepr*^*loxTB/loxTB*^, n = 6 for *Cre*^+^*Lepr*^+*/*+^, and n = 5 for *Cre*^*−*^*Lepr*^+*/*+^. In (**B**) and (**C**), n = 4 for *Cre*^+^*Lepr*^*loxTB/loxTB*^, n = 4 for *Cre*^*−*^*Lepr*^*loxTB/loxTB*^, n = 6 for *Cre*^+^*Lepr*^+*/*+^, and n = 6 for *Cre*^*−*^*Lepr*^+*/*+^. In (**D**), n = 4 for *Cre*^+^*Lepr*^*loxTB/loxTB*^, n = 4 for *Cre*^*−*^*Lepr*^*loxTB/loxTB*^, n = 5 for *Cre*^+^*Lepr*^+*/*+^, and n = 5 for *Cre*^*−*^*Lepr*^+*/*+^. In E–H, n = 4 for *Cre*^+^*Lepr*^*flox/flox*^ and n = 3 for *Cre*^*−*^*Lepr*^*flox/flox*^. Performed one-way ANOVA followed by Dunnett’s post-hoc test for *Lepr* reconstitution study and unpaired t-test for *Lepr* knockdown study. *p < 0.05, *Cre*^+^*Lepr*^*flox/flox*^ vs. *Cre*^*−*^*Lepr*^*flox/flox*^.

Adipose tissue cytokine expression was assessed to determine if alterations in pro-inflammatory (*Tnf* and *Il6*) and anti-inflammatory (*Arg1* and *Il10*) gene expression were associated with improvements in glycemic control in males with myeloid cell-specific *Lepr* reconstitution. Compared to wild-type controls, there were no statistically significant differences in *Tnf*, *Il6*, and *Arg1* expression (Fig. 11A–C), but there was a trend for *Il10* expression to be higher in mice with myeloid-cell specific *Lepr* reconstitution (p = 0.0774; Fig. 11D). To complement these determinations in the *Lepr* reconstitution colony, expression of pro-inflammatory and anti-inflammatory cytokines was also assessed in adipose tissue of males from the *Lepr* knockdown colony (Fig. 11E–H). Adipose tissue *Il6* mRNA was significantly decreased in mice with myeloid cell-specific *Lepr* knockdown relative to controls (p < 0.05; Fig. 11F).

## Discussion

We found that male mice with myeloid cell-specific *Lepr* reconstitution have diminished hyperglycemia in an age-dependent manner as well as better glucose tolerance compared to mice with whole-body transcriptional blockade of *Lepr*. Female mice with myeloid cell-specific *Lepr* reconstitution did not have ameliorated glucose metabolism, but there was a trend towards reduced hyperglycemia after a prolonged (16 h) fast. Reconstitution of *Lepr* did not significantly alter insulin sensitivity. The improved glycemic control in males with myeloid cell-specific *Lepr* reconstitution was associated with a trend for increased expression of the anti-inflammatory cytokine *Il10* in adipose tissue. We also found that male and female mice with myeloid cell-specific *Lepr* knockdown had similar body weight, blood glucose, plasma parameters, insulin sensitivity, and glucose tolerance to controls. Our results indicate that restoration of *Lepr* expression in cells of myeloid lineage has beneficial effects on glucose metabolism in male mice, but its knockdown does not have a significant impact. Since recombination was less than 100%, our results indicate that the remaining *Lepr* expression is sufficient for the maintenance of normal glucose metabolism in mice with *Lepr* knockdown.

Bone marrow transplantation using *db/db* and wild-type mice has been used to determine the role of immune cell leptin signaling on glucose metabolism and cytokine expression, yet results have been inconsistent and both innate and adaptive immune systems are affected in this model^20–23^. The reasons for these inconsistent results are unclear, but different study designs, including diets, and the invasiveness of irradiation could be possible explanations. The underlying mechanisms for the improved glucose metabolism in male mice with *Lepr* reconstitution may involve increased adipose expression of the anti-inflammatory cytokine *Il10*. Leptin directly increases IL-10 secretion by macrophages^8^ and administration of M2 macrophages (anti-inflammatory) to mice fed a high fat diet improves their glucose tolerance while also augmenting IL-10 levels in adipose tissue and the circulation^42^. Furthermore, IL-10 increases markers of M2 macrophages in adipose tissue^43^. Interestingly, we also found that adipose tissue *Il6* expression is decreased in mice with myeloid cell-specific *Lepr* knockdown. IL-6 can stimulate the bactericidicity of macrophages^44^ and it has already been demonstrated that macrophages from mice with myeloid cell-specific Leprb knockdown are functionally impaired in eradicating certain types of bacteria^26^. Hence, our results are consistent with reduced leptin signaling in macrophages causing impairments in their immunological action, possibly via diminished IL-6. Recently, Han et al.^45^ demonstrated that the source of IL-6 ultimately impacts its effects on glucose metabolism since adipocyte-specific *Il6* knockout mice, but not myeloid cell-specific *Il6* knockout mice, have improved insulin sensitivity, albeit mildly. Since we did not observe a phenotype in mice with myeloid cell-specific *Lepr* knockdown, it appears that the decrease in adipose tissue *Il6* mRNA levels we observed in mice with *Lepr* knockdown is due to diminished myeloid cell-derived *Il6* mRNA. In our study, adipose tissue *Il6* mRNA levels were approximately twofold lower in males with myeloid cell-specific *Lepr* reconstitution vs. males with global *Lepr* transcriptional blockade. Han et al.^45^ found that adipocyte-specific *Il6* knockout mice on high fat diet have decreased fasting blood glucose concentrations, while the opposite was found for myeloid cell-specific *Il6* knockout mice on high fat diet. Since we observed improved glycemic control in mice with myeloid cell-specific *Lepr* reconstitution (these mice have an obese phenotype similar to mice on high fat diet), it is possible that diminished adipocyte-derived *Il6* mRNA reduces adipose tissue *Il6* expression and thereby contributes to the amelioration in glucose metabolism.

Plasma insulin concentrations were not significantly different between *Cre*^+^*Lepr*^*loxTB/loxTB*^ and *Cre*^*−*^*Lepr*^*loxTB/loxTB*^ male mice, but the blunted blood glucose concentrations in *Cre*^+^*Lepr*^*loxTB/loxTB*^ mice during the recovery phase of the ITT (90 min and 120 min) suggests that counterregulatory hormone action, including glucagon and/or corticosterone, is diminished in male mice with *Lepr* reconstitution. Indeed, circulating glucagon concentrations are elevated in mice with whole-body *Lepr* transcriptional blockade (*Cre*^*−*^*Lepr*^*loxTB/loxTB*^) and in *db/db* mice, which have a comparable phenotype^46–48^. Interestingly, proopiomelanocortin (POMC) neuron-specific *Lepr* reconstitution improves glycemia, normalizes hepatic glucose output, and reduces plasma glucagon concentrations^48^. In our study, reduced circulating glucagon and consequently, increased insulin to glucagon ratio in the circulation may underlie the improved blood glucose concentrations and glucose tolerance observed in male mice with myeloid-cell specific *Lepr* reconstitution. In contrast to males, females with restored *Lepr* expression in myeloid cells had lower plasma insulin concentrations during the OGTT compared to females with global transcriptional blockade of *Lepr*, which could explain why females with *Lepr* reconstitution do not have improved glucose tolerance. Lastly, the increase in blood glucose concentrations in *Cre*^+^*Lepr*^*loxTB/loxTB*^ and *Cre*^*−*^*Lepr*^*loxTB/loxTB*^ mice at 24 h vs. 16 h of fasting could be associated with the circadian rhythm of circulating glucagon in rodents^49^.

To the best of our knowledge, three types of myeloid cell-specific *Lepr* knockdown mice have been reported in the literature: Metlakunta et al.^8^ generated mice with knockdown of all Lepr isoforms using *Lyz2Cre* mice, Scheller et al.^24^ and Mancuso et al.^26^ generated mice with knockdown of the Leprb isoform using *Lyz2Cre* mice, and Gao et al.^27^ created mice with knockdown of the Leprb isoform using *Cx3Cr1-Cre* mice. *Lyz2Cre* mice were also used in our study and we targeted Leprb for knockdown. Mice with myeloid cell-specific knockdown of all receptor isoforms gained more weight on standard chow^8^. Although neither Scheller et al.^25^, nor Mancuso et al.^26^, nor we found any differences in body weight in mice with myeloid cell-specific Leprb knockdown in younger male and female mice, Scheller et al.^25^ found that old (50 weeks of age) male mice tended to weigh more than controls. Mancuso et al.^26^ also found that pooled male and female mice with myeloid cell-specific Leprb knockdown had elevated circulating leptin levels compared to controls at 8 weeks old, but differences in plasma leptin levels across genotypes, for each sex, did not reach statistical significance at 12 weeks old in our study. Since the age of the mice was not specified by Metlakunta et al.^8^, we cannot comment if deletion of different Lepr isoforms affects phenotype. In mice with myeloid-cell specific knockdown of all Lepr isoforms, the ability of leptin to lower hepatic lipids is impaired, but glucose tolerance is similar to that of controls^8^. Our myeloid cell-specific Leprb knockdown male and female mice did not have different circulating FFAs, glucose, insulin sensitivity, and glucose tolerance compared to controls. Hence, *Lepr* knockdown in cells of myeloid lineage does not have a substantial impact on body weight regulation and glucose metabolism.

We used a genetic model obesity, namely *Lepr*^*loxTB/loxTB*^ mice, for various reasons. First, these mice have a similar phenotype to *db/db* mice, which is an extensively studied model of obesity, insulin resistance, hyperglycemia, hyperinsulinemia, hyperleptinemia, and hyperglucagonemia. Second, although an alternative to genetic obesity is high fat diet-induced obesity, mice lacking all isoforms of the leptin receptor in myeloid cells, generated using *Lyz2Cre* mice, have already been studied in the context of high fat diet feeding^8^. In that study by Metlakunta et al., on a high fat diet, mice with myeloid cell-specific *Lepr* knockdown had diminished body fat content, but similar body weight and glucose tolerance, to control mice. It is unclear if the glucose tolerance test results are in line with ours because the sex of the mice was not reported. However, our results are similar in that the body weight of mice with myeloid cell-specific *Lepr* reconstitution was comparable to the body weight of mice with global transcriptional blockade of *Lepr* (*Lyz2Cre*^*−*^*Lepr*^*loxTB/loxTB*^). The exception was in older age, when male *Lyz2Cre*^*−*^*Lepr*^*loxTB/loxTB*^ mice were slightly lighter than male *Lyz2Cre*^+^*Lepr*^*loxTB/loxTB*^ mice, likely as a result of prolonged poorer glycemic control in the former. Third, a high fat diet regimen may not induce hyperglycemia, thereby making the discovery of possible differences in glucose metabolism challenging and limiting the potential applicability of the results to humans with uncontrolled obesity-associated diabetes. Fourth, the outcome of high fat diet studies may depend on diet composition and duration, in addition to age of the mice. Thus, genetic models of obesity likely minimize confounding variables, which may be interesting in their own right, but require a more complex study design and analysis.

*Lyz2Cre* mice are commonly used to study myeloid cell lineage and macrophage signaling using Cre-lox methodology. Cre-mediated excision with this transgene is limited to the innate immune system and also occurs peripherally in neutrophils and centrally in microglia^30,31,50^. We found approximately 75% *Lepr*^*flox*^ recombination in bone marrow-derived macrophages. Bone marrow-derived macrophages are usually used to determine Cre-mediated excision in *Lyz2Cre* mice^24,29^, but Cre-mediated excision in myeloid cells is commonly either not reported or not quantified in studies, thereby making the recombination we obtained difficult to compare. Results from studies using reporter mice indicate that *Cx3Cr1-Cre* can induce recombination not only in macrophages, but also microglia, granulocytes, natural killer cells and even the adaptive immune system, namely T lymphocytes and B lymphocytes (The Jackson Laboratory). Gao et al.^27^ used *Cx3Cr1-Cre* mice to knock down Leprb and the increased body weight they found in mice as early as 10 weeks old was associated with diminished hypothalamic POMC neurons and morphological changes in microglia, indicating an important role for microglia in the mouse model. We did not observe changes in body weight, but we cannot exclude that Lepr signalling in microglia was affected in our models.

In conclusion, male mice with myeloid cell-specific *Lepr* reconstitution have improved glucose metabolism, yet are still hyperglycemic compared to lean controls. While there is debate in the literature whether leptin can adversely affect inflammatory status of immune cells, and consequently, glucose metabolism, our results indicate that leptin signaling in the innate immune system has beneficial metabolic effects, at least in male mice.

## Supplementary Information

Supplementary Information.

## Data Availability

The datasets generated during and/or analysed during the current study are available from the corresponding author on reasonable request.
