# Supplementary material for: Role of myeloid cell leptin signaling in the regulation of glucose metabolism
Source: Sci Rep. 2021 Sep 15;11:18394. doi: 10.1038/s41598-021-97549-0 (PMC8443652; doi:10.1038/s41598-021-97549-0)
Supplement: Supplementary file 1 — Supplementary Information. [file 41598_2021_97549_MOESM1_ESM.docx]

**SUPPLEMENTARY INFORMATION**

**Role of myeloid cell leptin signaling in the regulation of glucose metabolism**

Sandra Pereira^1^, Daemon L. Cline^1^, Melissa Chan^1^, Kalin Chai^1^, Ji Soo Yoon^1^, Shannon M. O'Dwyer^1^, Cara E. Ellis^1^, Maria M. Glavas^1^, Travis D. Webber^1^, Robert K. Baker^1^, Suheda Erener^1^, Scott D. Covey^2^, Timothy J. Kieffer^1,3,4^

^1^ Department of Cellular and Physiological Sciences, University of British Columbia, 2350 Health Sciences Mall, Vancouver, BC, V6T 1Z3, Canada

^2^ Department of Biochemistry and Molecular Biology, University of British Columbia, 2350 Health Sciences Mall, Vancouver, BC, V6T 1Z3, Canada

^3^ Department of Surgery, University of British Columbia, 2775 Laurel Street, Vancouver, BC, V5Z 1M9, Canada

^4^ School of Biomedical Engineering, University of British Columbia, 251-2222 Health Sciences Mall, Vancouver, BC, V6T 1Z3, Canada

**SUPPLEMENTARY METHODS**

*Genotyping*

Primers used for genotyping:

**Lyz2Cre genotyping:**

Endpoint PCR:

*Lyz2Cre:*

Forward primer (Lyz2Cre-F): CTT GGG CTG CCA GAA TTT CTC

Reverse primer (CreRX2): CCT GAT CCT GGC AAT TTC GG

*Wild-type allele:*

Forward primer (Lyz2Cre-F): CTT GGG CTG CCA GAA TTT CTC

Reverse primer (Lyz2Cre-R1): TTA CAG TCG GCC AGG CTG AC

qPCR:

*Lyz2Cre:*

Forward primer (qLyz2Cre-F1): GAC TTG GAG GAT GCT TAA ATA G

Reverse primer (qLyz2Cre-R1): GTC AGT AAA TTG GAC ACC TTC

Probe (qLyz2Cre-P1): TCC AGT CAC CAT GCC CAA GAA GAA GA

*Wild-type allele:*

Forward primer (qLyz2-F1): AGG CAT GCT TTC TCT AGT C

Reverse primer (qLyz2-R1): GCA AAC TCA CAA CGT TCA TA

Probe (qLyz2-P1): ACT CCT CCT GCT TTC TGT CAC TGC

**Leprflox genotyping:**

Endpoint PCR:

*Lepr^flox^ and Lepr^+^:*

Forward primer (LepRF): ACACCACACTGTTGAGACACC

Reverse primer (LepRR): CATTTGATTCCACAAAGTGTTCCCTAAAC

qPCR :

*Lepr^flox^*:

Forward primer (Lepr Ex17-F): TTA ACC CAG ATA TCG AAT TCC T

Reverse primer (Lepr Ex17-R): GAG AAC ATG AAC ACA ACA ACA

Probe (Lepr Ex17-HEX): CCC GAT TTC GAA CCC GAC TCT AGA CT

*Lepr^+^*:

Forward primer (qLepr(WT)-F1): CTG GGT GAT CTC ACA CAT AC

Reverse primer (qLepr(WT)-R1): CAT AAG TCC ACG GGA TAT GG

Probe (qLepr(WT)-P1): AGT GAG GAG GGA AGA CGT TAT AAT CT

**LeprloxTB genotyping:**

qPCR:

*Lepr^loxTB^*:

Forward primer (qLeprNull-F2): CTG CAT TCT AGT TGT GGT TTG

Reverse primer (qLeprNull-R2): GTC TCA TGA GCG GAT ACA TAT T

Probe (qLeprNull-P2): TCA TGT CTG GAT CGC TTA GGT GGC A

*Lepr^+^*:

Forward primer (qLepr(WT)-F2): GTG AGA TCA TGA GAC CCT AAA

Reverse primer (qLepr(WT)-R2): GGA ACT CAA GAC CAT CTA TCA

Probe (qLepr(WT)-P2): TTC TGA ATT GGT GTC CCT GGA GCC

**SUPPLEMENTARY RESULTS**

**Supplementary Figure S1.** Genotyping results for ear notch samples from the myeloid cell-specific *Lepr* reconstitution colony, which is referred to as “MacroLeprEXP” in the online database from our animal facility. “Sample” refers to the ear notch from a given mouse and each mouse was assigned a number by our animal facility staff. **A:** Lyz2Cre genotyping results using qPCR, including control samples. Control WT, *Lyz2Cre^-^*; Control HET, hemizygous *Lyz2Cre^+^*; Control Lyz2Cre, homozygous *Lyz2Cre^+^*. **B:** LeprloxTB genotyping results using qPCR, including control samples. Control WT, *Lepr^+/+^*; Control Het, *Lepr^loxTB/+^*; Control NULL, *Lepr^loxTB/loxTB^*. Some mice did not belong to the myeloid cell-specific *Lepr* reconstitution colony and therefore the identity of their strain was removed. **C:** Summary of genotyping results of mice from the myeloid cell-specific *Lepr* reconstitution colony based on data from A and B.

**A: Lyz2Cre qPCR**


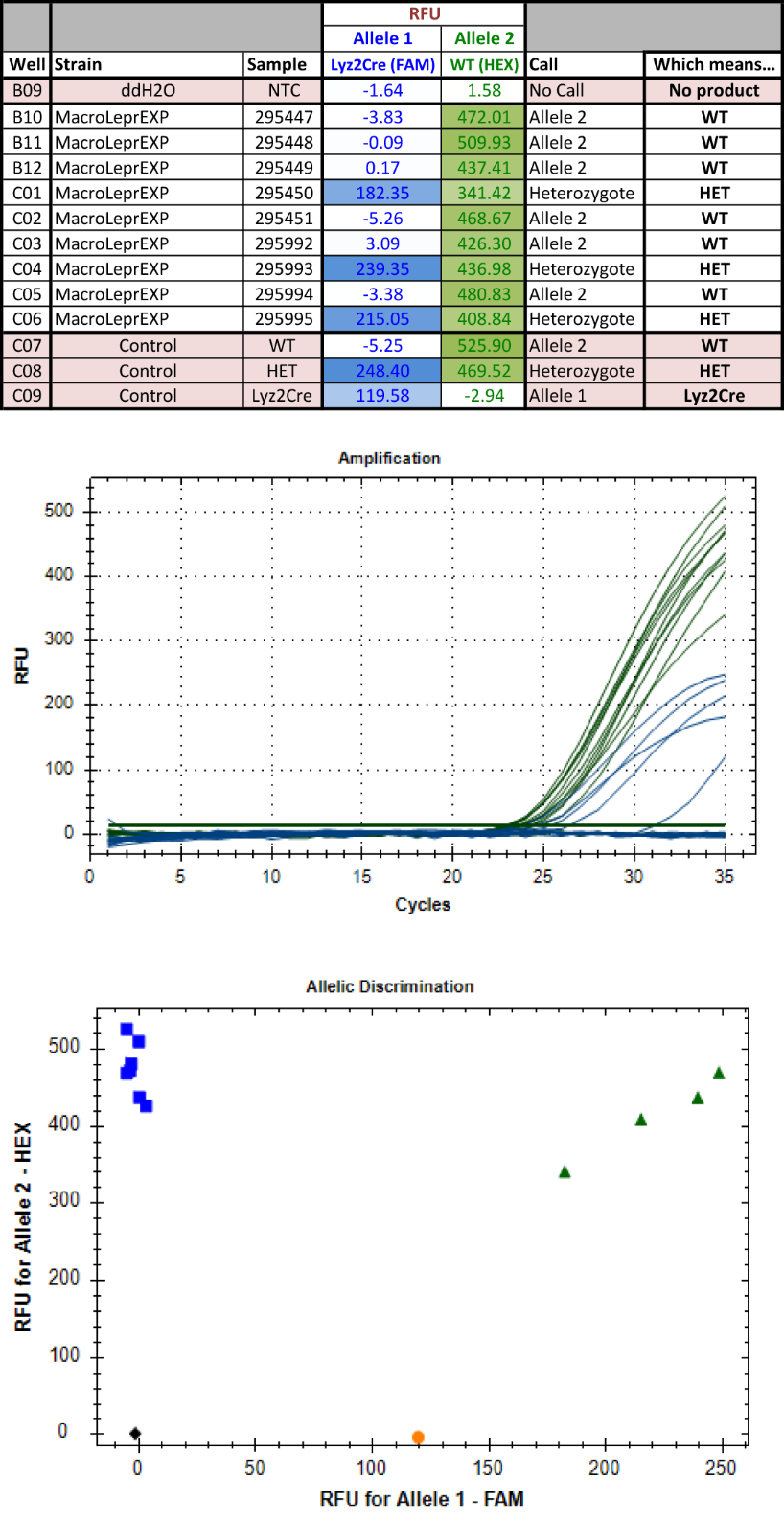


**B: LeprloxTB qPCR**


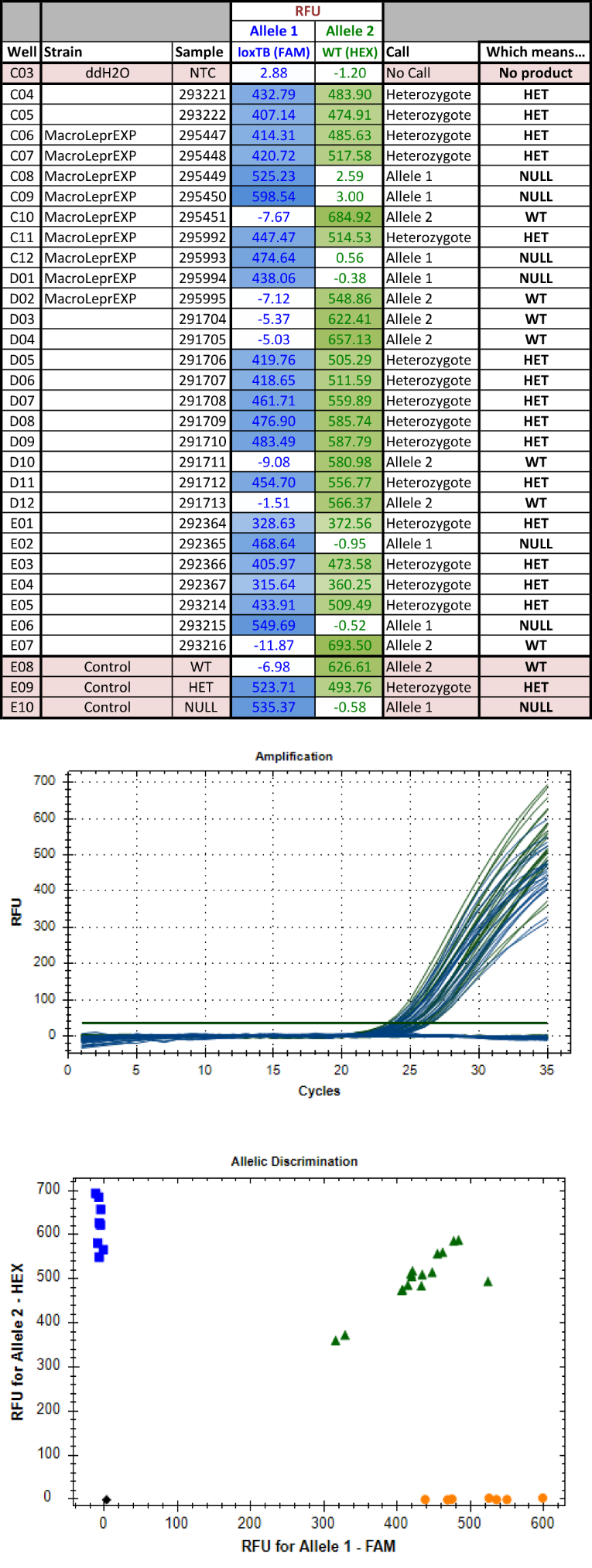


**C: Summary of genotyping results from A and B**

All *Lyz2Cre^+^* mice in this table are hemizygous for *Cre*

| **Sample** | **Genotype** |
| --- | --- |
| 295447 | *Lyz2Cre^-^Lepr^loxTB/+^* |
| 295448 | *Lyz2Cre^-^Lepr^loxTB/+^* |
| 295449 | *Lyz2Cre^-^Lepr^loxTB/loxTB^* |
| 295450 | *Lyz2Cre^+^Lepr^loxTB/loxTB^* |
| 295451 | *Lyz2Cre^-^Lepr^+/+^* |
| 295992 | *Lyz2Cre^-^Lepr^loxTB/+^* |
| 295993 | *Lyz2Cre^+^Lepr^loxTB/loxTB^* |
| 295994 | *Lyz2Cre^-^Lepr^loxTB/loxTB^* |
| 295995 | *Lyz2Cre^+^Lepr^+/+^* |

**Supplementary Figure S2.** Genotyping results for ear notch samples from the myeloid cell-specific *Lepr* knockdown colony. “Sample” refers to the ear notch from a given mouse and each mouse was assigned a number by our animal facility staff. **A:** Lyz2Cre genotyping results using endpoint PCR, including DNA ladder and control samples. Lyz2Cre genotyping controls - WT, *Lyz2Cre^-^*; HET, hemizygous *Lyz2Cre^+^*; CRE, homozygous *Lyz2Cre^+^*. **B:** Leprflox genotyping results using endpoint PCR, including DNA ladder and control samples. Leprflox genotyping controls - WT, *Lepr^+/+^*; HET, *Lepr^flox/+^*; FLOX, *Lepr^flox/flox^*. **C:** Lyz2Cre genotyping results using qPCR, including control samples. Control WT, *Lyz2Cre^-^*; Control HET, hemizygous *Lyz2Cre^+^*; Control Lyz2Cre, homozygous *Lyz2Cre^+^*. The strain “Lyz2-Cre”, as it is called in the online database from our animal facility, is the myeloid cell-specific *Lepr* knockdown colony. **D:** Leprflox genotyping results using qPCR, including control samples. Control WT, *Lepr^+/+^*; Control Het, *Lepr^flox/+^*; Control FLOX, *Lepr^flox/flox^*. The strain “Lyz2-Cre”, as it is called in the online database from our animal facility, is the myeloid cell-specific *Lepr* knockdown colony. **E:** Summary of genotyping results of mice from the myeloid cell-specific *Lepr* knockdown colony based on data from A, B, C, and D.

**A: Lyz2Cre endpoint PCR**

**
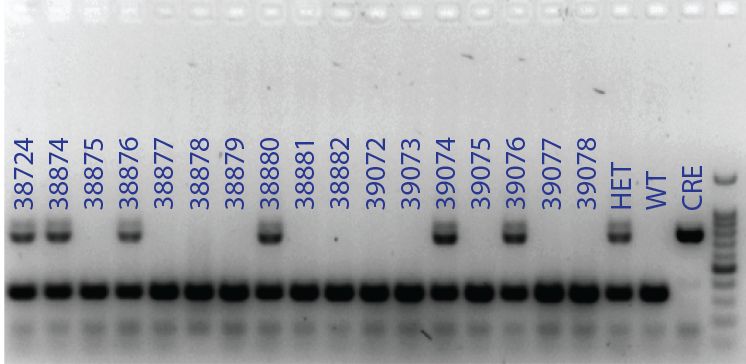
**

**bp**

800

700

600

←500 (darkest band)

400

300

200

**B: Leprflox endpoint PCR**

**
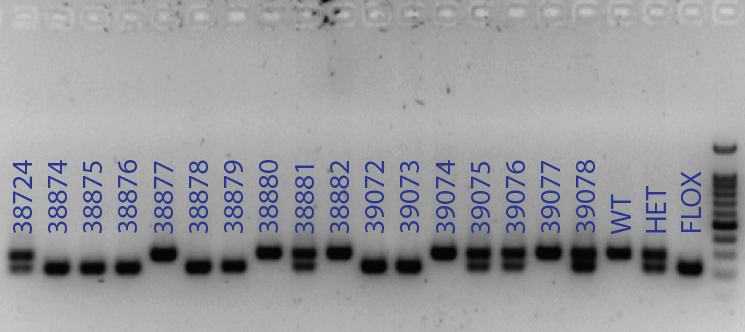
**

**C: Lyz2Cre qPCR** (the strain “Lyz2-Cre”, as it is called in the online database from our animal facility, is the myeloid cell-specific *Lepr* knockdown colony)


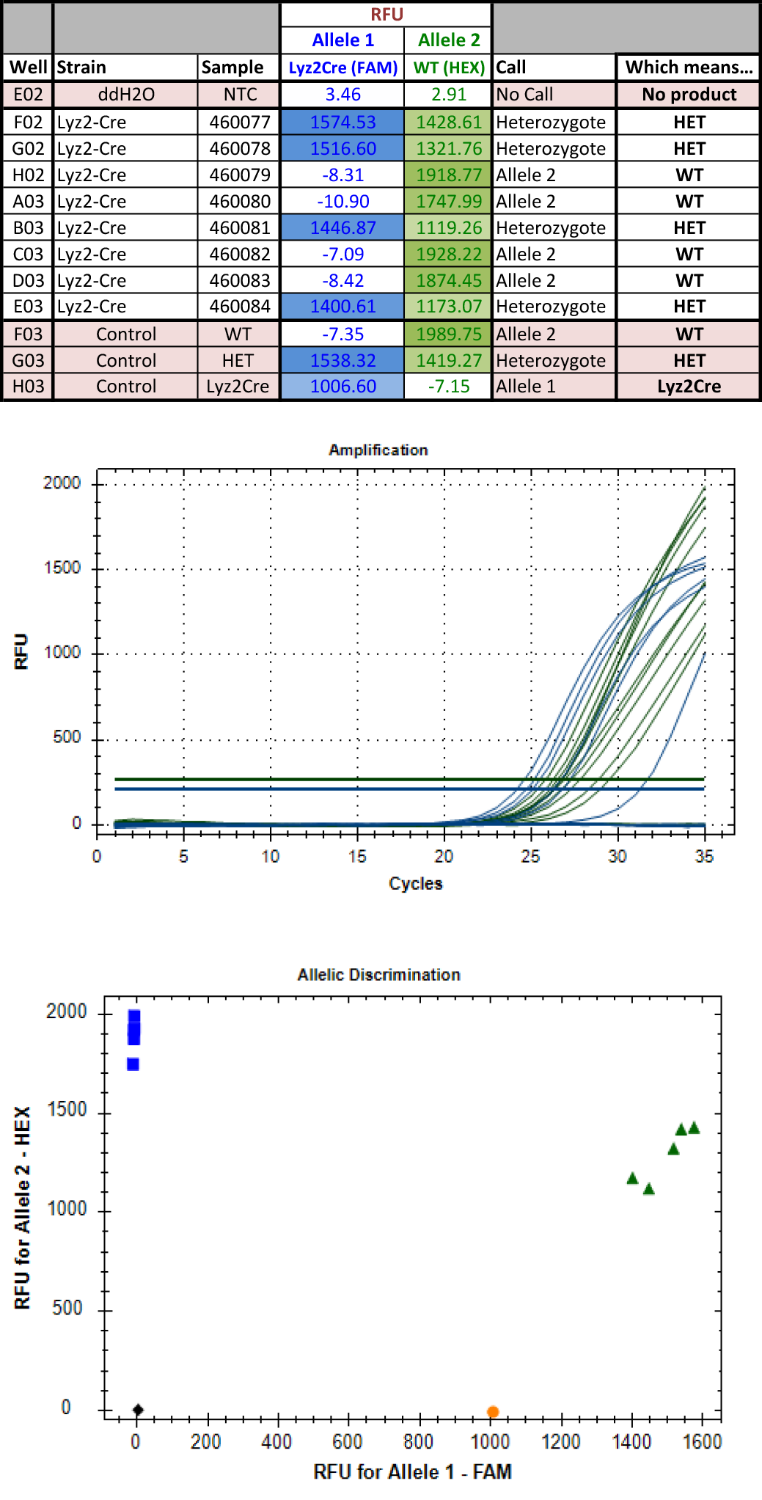


**D: Leprflox qPCR** (the strain “Lyz2-Cre”, as it is called in the online database from our animal facility, is the myeloid cell-specific *Lepr* knockdown colony)

**
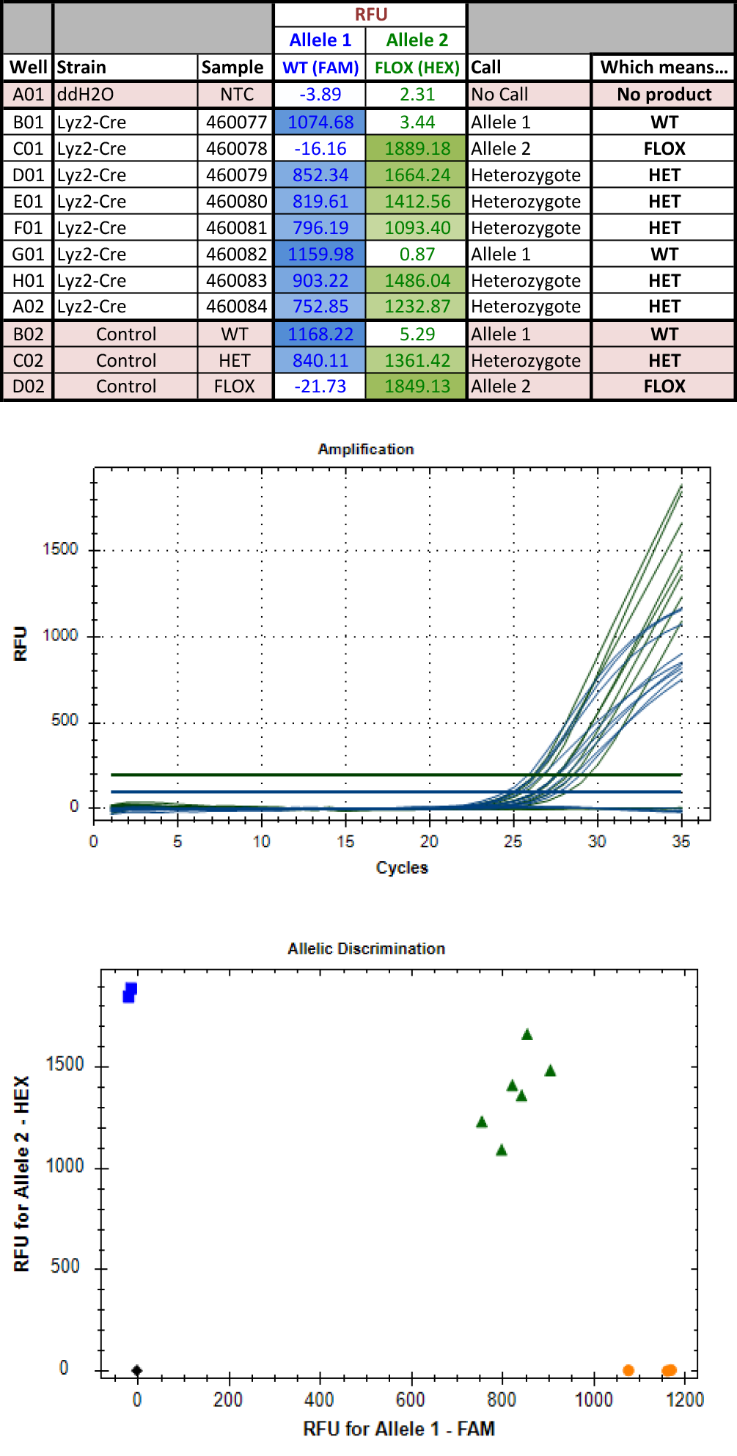
**

**E: Summary of genotyping results from A, B, C, and D**

All *Lyz2Cre^+^* mice in this table are hemizygous for *Cre*

| **Sample** | **Genotype** |
| --- | --- |
| 38724 | *Lyz2Cre^+^Lepr^flox/+^* |
| 38874 | *Lyz2Cre^+^Lepr^flox/flox^* |
| 38875 | *Lyz2Cre^-^Lepr^flox/flox^* |
| 38876 | *Lyz2Cre^+^Lepr^flox/flox^* |
| 38877 | *Lyz2Cre^-^Lepr^+/+^* |
| 38878 | *Lyz2Cre^-^Lepr^flox/flox^* |
| 38879 | *Lyz2Cre^-^Lepr^flox/flox^* |
| 38880 | *Lyz2Cre^+^Lepr^+/+^* |
| 38881 | *Lyz2Cre^-^Lepr^flox/+^* |
| 38882 | *Lyz2Cre^-^Lepr^+/+^* |
| 39072 | *Lyz2Cre^-^Lepr^flox/flox^* |
| 39073 | *Lyz2Cre^-^Lepr^flox/flox^* |
| 39074 | *Lyz2Cre^+^Lepr^+/+^* |
| 39075 | *Lyz2Cre^-^Lepr^flox/+^* |
| 39076 | *Lyz2Cre^+^Lepr^flox/+^* |
| 39077 | *Lyz2Cre^-^Lepr^+/+^* |
| 39078 | *Lyz2Cre^-^Lepr^flox/+^* |
| 460077 | *Lyz2Cre^+^Lepr^+/+^* |
| 460078 | *Lyz2Cre^+^Lepr^flox/flox^* |
| 460079 | *Lyz2Cre^-^Lepr^flox/+^* |
| 460080 | *Lyz2Cre^-^Lepr^flox/+^* |
| 460081 | *Lyz2Cre^+^Lepr^flox/+^* |
| 460082 | *Lyz2Cre^-^Lepr^+/+^* |
| 460083 | *Lyz2Cre^-^Lepr^flox/+^* |
| 460084 | *Lyz2Cre^+^Lepr^flox/+^* |
